# Comparative Effectiveness of Diversion of Cerebrospinal Fluid for Children With Severe Traumatic Brain Injury

**DOI:** 10.1001/jamanetworkopen.2022.20969

**Published:** 2022-07-08

**Authors:** Michael J. Bell, Bedda L. Rosario, Patrick M. Kochanek, P. David Adelson, Kevin P. Morris, Alicia K. Au, Michelle Schober, Warwick Butt, Richard J. Edwards, Jerry Zimmerman, Jose Pineda, Truc M. Le, Nathan Dean, Michael J. Whalen, Anthony Figaji, James Luther, Sue R. Beers, Deepak K. Gupta, Jessica Carpenter, Sandra Buttram, Stephen R. Wisniewski

**Affiliations:** 1 Division of Critical Care Medicine, Department of Pediatrics, Children’s National Medical Center, Washington, District of Columbia; 2 Department of Epidemiology, University of Pittsburgh, Pittsburgh, Pennsylvania; 3 Department of Critical Care Medicine, University of Pittsburgh School of Medicine, Pittsburgh, Pennsylvania; 4 Barrow Neurological Institute at Phoenix Children’s Hospital, Phoenix, Arizona; 5 Division of Pediatric Critical Care, Birmingham Children’s Hospital NHS Foundation, Birmingham, United Kingdom; 6 Division of Pediatric Critical Care Medicine, University of Utah, Salt Lake City; 7 Division of Pediatric Critical Care Medicine, The Royal Children’s Hospital, Melbourne, Australia; 8 Division of Paediatric Neurosurgery, Bristol Royal Hospital for Children, Bristol, United Kingdom; 9 Division of Pediatric Critical Care Medicine, Harborview Medical Center, Seattle, Washington; 10 Division of Pediatric Critical Care Medicine, St Louis Children’s Hospital, St Louis, Missouri; 11 Division of Critical Care Medicine, Department of Pediatrics, Vanderbilt University, Nashville, Tennessee; 12 Division of Pediatric Critical Care Medicine, Massachusetts General Hospital, Boston; 13 Department of Neurosurgery, Red Cross War Memorial Children’s Hospital, Cape Town, South Africa; 14 Department of Psychiatry, University of Pittsburgh, Pittsburgh, Pennsylvania; 15 Department of Neurosurgery, All India Institute of Medical Sciences, New Delhi, India; 16 University of Maryland Children’s Hospital, Baltimore

## Abstract

**Importance:**

Diversion of cerebrospinal fluid (CSF) has been used for decades as a treatment for children with severe traumatic brain injury (TBI) and is recommended by evidenced-based guidelines. However, these recommendations are based on limited studies.

**Objective:**

To determine whether CSF diversion is associated with improved Glasgow Outcome Score–Extended for Pediatrics (GOS-EP) and decreased intracranial pressure (ICP) in children with severe TBI.

**Design, Setting, and Participants:**

This observational comparative effectiveness study was performed at 51 clinical centers that routinely care for children with severe TBI in 8 countries (US, United Kingdom, Spain, the Netherlands, Australia, New Zealand, South Africa, and India) from February 2014 to September 2017, with follow-up at 6 months after injury (final follow-up, October 22, 2021). Children with severe TBI were included if they had Glasgow Coma Scale (GCS) scores of 8 or lower, had intracranial pressure (ICP) monitor placed on-site, and were aged younger than 18 years. Children were excluded if they were pregnant or an ICP monitor was not placed at the study site. Consecutive children were screened and enrolled, data regarding treatments were collected, and at discharge, consent was obtained for outcomes testing. Propensity matching for pretreatment characteristics was performed to develop matched pairs for primary analysis. Data analyses were completed on April 18, 2022.

**Exposures:**

Clinical care followed local standards, including the use of CSF diversion (or not), with patients stratified at the time of ICP monitor placement (CSF group vs no CSF group).

**Main Outcomes and Measures:**

The primary outcome was GOS-EP at 6 months, while ICP was considered as a secondary outcome. CSF vs no CSF was treated as an intention-to-treat analysis, and a sensitivity analysis was performed for children who received delayed CSF diversion.

**Results:**

A total of 1000 children with TBI were enrolled, including 314 who received CSF diversion (mean [SD] age, 7.18 [5.45] years; 208 [66.2%] boys) and 686 who did not (mean [SD] age, 7.79 [5.33] years; 437 [63.7%] boys). The propensity-matched analysis included 98 pairs. In propensity score–matched analyses, there was no difference between groups in GOS-EP (median [IQR] difference, 0 [−3 to 1]; *P* = .08), but there was a decrease in overall ICP in the CSF group (mean [SD] difference, 3.97 [0.12] mm Hg; *P* < .001).

**Conclusions and Relevance:**

In this comparative effectiveness study, CSF diversion was not associated with improved outcome at 6 months after TBI, but a decrease in ICP was observed. Given the higher quality of evidence generated by this study, current evidence-based guidelines related to CSF diversion should be reconsidered.

## Key Points

**Question:**

Is cerebrospinal fluid (CSF) diversion associated with improved outcomes or intracranial pressure in children with severe traumatic brain injury (TBI)?

**Findings:**

In this comparative effectiveness study of 1000 children with severe TBI, there was no association between CSF diversion and Glasgow Outcome Score–Extended for Pediatrics at 6 months after injury in propensity-matched participants. However, CSF diversion was associated with decreased intracranial pressure in the propensity-matched participants.

**Meaning:**

These findings suggest that the current evidenced-based guidelines that support CSF diversion as a first-line therapy for TBI in children should be reconsidered.

## Teaser

This comparative effectiveness study assesses the associations of cerebrospinal fluid diversion with Glasgow Outcome Score–Extended for Pediatrics and decreased intracranial pressure in children with severe traumatic brain injury.

## Introduction

Drainage of cerebrospinal fluid (CSF) for neurosurgical emergencies, called *CSF diversion*, was first described in 1774,^1,2^ and it remains one of the most common neurosurgical procedures for neurological conditions, including traumatic brain injury (TBI).^3^ Removing CSF can lead to decreases in intracranial pressure (ICP),^4^ and uncompensated increases in ICP can impair cerebral perfusion and lead to adverse outcomes. Placement of devices to drain CSF can allow for ICP measurement at the bedside, while intraparenchymal ICP catheters are also available to measure ICP without performing CSF diversion. The risk-benefit decision for which device to place is central to contemporary neurocritical care.

Seminal studies demonstrated associations between intracranial hypertension and mortality after TBI, and these studies concurrently led to recommendations for CSF diversion as a treatment strategy.^5,6,7,8^ Subsequently, 7 versions of evidenced-based guidelines for severe TBI for adults^9,10,11,12^ and children^13,14,15^ suggest use of CSF diversion. The current recommendation for children is that that CSF drainage through an external ventricular drain (EVD) is suggested to manage increased ICP based on data from 56 children in 3 published reports.^15^ Because of the weakness of the evidence,^16^ we sought to determine the associations between CSF diversion and neurological outcomes in children with severe TBI. After conducting a survey of 32 pediatric centers to determine their current practices related to CSF diversion, we found substantial intercenter and intracenter variability, strongly suggesting a lack of equipoise that would imperil a randomized study design.^17^ Therefore, we conducted an observational comparative effectiveness study using propensity matching to test the primary hypothesis that CSF diversion is associated with improved outcomes after severe TBI. As a secondary hypothesis, we sought to determine if CSF diversion in these matched patients is associated with decreased ICP.

## Methods

### Study Design

The Approaches and Decisions for Acute Pediatric TBI (ADAPT) trial was an observational comparative effectiveness study funded by a cooperative agreement with the US National Institute of Neurological Disorders and Stroke). The study included sites in the US, United Kingdom, Spain, the Netherlands, India, South Africa, Australia, and New Zealand (eTable 1 in Supplement 1). All sites obtained institutional human research review board approval (institutional review board or equivalent), and the University of Pittsburgh received institutional review board approval to coordinate the study. All sites were permitted to perform data collection, including therapies that were administered as standard of care prior to informed consent. Families were approached for informed consent for outcome assessments at the time of ICU discharge. Therefore, this cohort represents consecutive children meeting inclusion and exclusion criteria. We followed the Strengthening the Reporting of Observational Studies in Epidemiology (STROBE) reporting guideline.

The inclusion criteria were age younger than 18 years, diagnosis of TBI, CP monitor placed, and Glasgow Coma Scale (GCS) score of 8 or less at the time of monitor placement. Because we intended ADAPT to inform the evidenced-based guidelines for severe TBI and many of the guideline topics are related to ICP-based therapies, we limited inclusion to children who had ICP monitors placed as part of their clinical care. Importantly, sites chose the ICP monitoring modality (EVD devices that measure ICP and perform CSF diversion or intraparenchymal devices that measure ICP but cannot perform CSF diversion) at their discretion. We recorded the GCS score at the time of ICP monitoring to ensure that all participants met the current definition for severe TBI (GCS score ≤8). Exclusion criteria were pregnancy (to avoid potential confounding of therapies for TBI with pregnancy-related concerns) and ICP monitor placement at another institution (to ensure data availability for early therapies administered).

Data collection occurred during the prehospital phase (defined as the time of injury until arrival at the study site), resuscitation phase (defined as arrival time at study hospital until ICP monitor placement), ICP therapy phase (defined as time of ICP monitor placement until either 7 days or ICP monitor removal), hospital phase (defined as the end of the ICP monitoring phase until hospital discharge), and follow-up phase (defined as 6 months after ICP monitor placement time), as previously reported.^18,19,20^ Patient demographics, injury details, imaging findings, severity of illness scores, and prehospital and resuscitation events were collected consistent with the National Institute of Neurological Disorders and Stroke TBI Common Data Elements (CDE)^21,22,23^ (eTable 2 in Supplement 1). To assess if CSF strategies were associated with complications, we collected data on general, cardiovascular, respiratory, and neurological complications after ICP monitor placement (eTable 3 in Supplement 1). A variety of methods were used to determine the Glasgow Outcome Score–Extended for Pediatrics (GOS-EP) scores from study participants. From consented participants, site personnel assessed GOS-EP by phone, as previously described.^24^ If consented participants were not available or parents refused outcomes assessments but allowed continuing review of medical information, then medical records were reviewed for outcomes determination.

Sites recorded information regarding all neurosurgical procedures that occurred, including insertion of an EVD. Sites recorded the strategy of CSF diversion (CSF diversion group vs no CSF diversion group) at the time of ICP monitor placement and this strategy was used as an intention-to-treat decision for this analysis (eTable 2 in Supplement 1). Participants who received continuous or intermittent CSF diversion were considered as receiving CSF diversion for this analysis. If participants who were treated without CSF diversion at the time of ICP monitor placement received CSF diversion later in their hospital course, this information was collected, but the participants remained in the no CSF diversion group for the primary analyses. A sensitivity analysis was performed to determine if delayed CSF diversion was associated with different outcomes. Similar results would indicate that the results are robust and not impacted by the time with the ICP monitor was placed. Hourly ICP readings were recorded, and these values were used for the secondary analysis.

### Statistical Methods

Baseline characteristics were summarized for each group and compared with independent sample *t* test (or Wilcoxon Rank Sum test) or χ^2^ test (or Fisher exact test). Bivariate regression (proportional odds, logistic, and Cox proportional hazards) models were used to assess the strength of the association of CSF diversion with each outcome (GOS-EP, mortality, time to death, and complications). A concern with observational studies is that there are confounders and biases associated with treatment allocation (eg, higher severity of injury may be related to receiving the intervention). Propensity score analysis was used to reduce the potential impact of confounding and selection bias effects. The propensity score is the probability of treatment assignment conditional on observed baseline characteristics. We estimated the propensity scores using the generalized boosted models (GBM) approach^25,26^ and considered all characteristics collected. We used the Toolkit for Weighting and Analysis of Nonequivalent Groups (twang) software package (RAND)^27^ and SAS macros (SAS Institute) to estimate and evaluate the propensity scores. Propensity-matched analysis with a 1:1 ratio was used to compare the outcomes of interest. We allowed a maximum of 3 splits for each tree in the model, allowing for 3-way interactions among all covariates to be considered. The shrinkage parameter was set to 0.005 to ensure a smooth fit. Balance tables and plots were used to assess the quality of the propensity scores, to compare the propensity score distributions between treatment groups, and to evaluate the common support. From the GBM approach, a propensity score indicating the probability of CSF diversion strategy (yes or no) given observed baseline characteristics was obtained for each participant. Caliper matching without replacement within specified calipers of the logit of the propensity score was used for propensity score matching.^28,29^

The primary outcome of the study was GOS-EP scores at 6 months. Other end points were evaluated as exploratory, and no adjustments for multiple comparisons were made. For our primary outcome of propensity-matched participants, the signed rank test was used to test the association of group with GOS-EP score. An ordinal regression model was then used to estimate the association of CSF diversion with GOS-EP score after controlling for characteristics not balanced through matching.^30^ After propensity matching, a conditional logistic regression model was used to assess the association of propensity-matched CSF group with mortality and complications. A conditional Cox proportional hazards model was used to assess the association of propensity-matched CSF group with time to death. Baseline characteristics that were unbalanced after matching by propensity score were included in the models. As a sensitivity analysis, a combination of inverse probability of received treatment weighting and unbalanced covariate adjustment (ie, doubly robust estimation^31^) was performed if the propensity scores were not able to completely balance participants’ characteristics for all outcomes. A similar analysis was performed as a sensitivity analysis to assess the effect of including children with delayed CSF diversion in the CSF diversion group. In an effort to broaden the number of participants included in the matched analysis, an additional sensitivity analysis was conducted excluding a subsample of participants not likely to receive the intervention. A classification tree model was used to identify subgroups who were not likely to receive CSF diversion. The independent variables in the classification tree were those that were included in the primary propensity score model. A propensity model was then fit using the independent variables included in the propensity score from the primary analysis. This analysis also resulted in a limited number of matched pairs. To increase the area of common support and number of matched pairs, the propensity model was restricted to characteristics that were associated with both the outcome and the exposure.

A longitudinal mixed effects regression model was used to compare the mean ICP over time between groups. The model included a main fixed effect for and indicator of CSF diversion therapy and a random effect for matched pair. All tests were 2-sided, and the significance level was set at *P* = .05. All analyses were conducted using SAS software version 9.3 (SAS Institute). Data were analyzed from [placeholder] to [placeholder].

## Results

**Figure 1.**
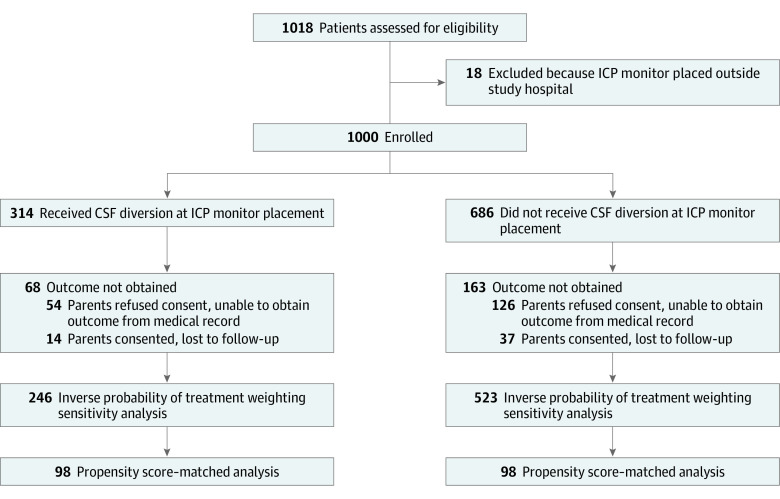
Participant Recruitment Flowchart Participants were stratified based on decisions regarding cerebrospinal fluid (CSF) diversion and were enrolled prior to obtaining consent for outcomes. ICP indicates intracranial pressure.

A total of 1018 children were screened for inclusion, and 18 children were excluded for having an ICP monitor placed outside of a study site (Figure 1). A total of 1000 children with TBI were enrolled, including 314 who received CSF diversion (mean [SD] age, 7.18 [5.45] years; 208 [66.2%] boys) and 686 who did not (mean [SD] age, 7.79 [5.33] years; 437 [63.7%] boys) (Table 1). The number of participants for whom outcomes could not be determined was similar between groups (Figure 1) yielding 246 participants in the CSF diversion group and 523 participants in the no CSF diversion group available for analysis. After propensity matching, 98 matched pairs were identified. Comparisons of characteristics in the overall cohort and the matched sample are summarized in Table 1. For the overall cohort, an unadjusted bivariate ordinal logistic regression model demonstrated that CSF diversion was associated with worse GOS-EP scores (odds ratio [OR], 1.36 [95% CI, 0.95-1.96]) and increased mortality at 28 days (OR, 1.26 [95% CI, 0.85-1.86]) (Table 2). After adjustment for factors not balanced between groups (Hispanic ethnicity, pupil size, partial pressure of blood oxygen monitor, decompressive craniectomy, extra-axial hematoma, intracranial hemorrhage, pediatric hospital, electronic health record, and site), these associations were not observed (GOS-EP: adjusted OR, 1.18 [95% CI, 0.71-.95]; mortality: adjusted OR, 0.74 [95% CI, 0.42-1.31]) (Table 2).

**Table 1.** Baseline Participant Characteristics

Characteristic	Full sample			Matched sample^a^		
	CSF Diversion, No. (%)		*P* value	CSF Diversion, No. (%)		P value
	Yes (n = 314)	No (n = 686)		Yes (n = 98)	No (n = 98)	
Age, y, mean (SD)	7.18 (5.45)	7.79 (5.33)	.10	6.69 (5.34)	7.33 (5.58)	.42
Sex						
Girls	106 (33.8)	249 (36.3)	.44	33 (33.7)	38 (38.8)	.46
Boys	208 (66.2)	437 (63.7)		65 (66.3)	60 (61.2)	
Primary race						
Black	77 (26.5)	136 (21.7)	<.001	24 (27.0)	24 (25.5)	.03
White	191 (65.6)	370 (59.0)		53 (59.6)	67 (71.3)	
Other^b^	23 (7.9)	121 (19.3)		12 (13.5)	3 (3.2)	
Hispanic ethnicity^c^	48 (19.1)	63 (17.7)	.65	8 (12.5)	23 (26.7)	.03
Cause of injury						
Motor vehicle	158 (50.3)	399 (58.2)	<.001	52 (53.1)	52 (53.1)	.64
Fall	46 (14.6)	135 (19.7)		16 (16.3)	21 (21.4)	
Homicide/assault	60 (19.1)	87 (12.7)		22 (22.4)	16 (16.3)	
Other	50 (15.9)	65 (9.5)		8 (8.2)	9 (9.2)	
Type of injury						
Open	43 (13.7)	53 (7.7)	.002	10 (10.2)	9 (9.2)	.81
Closed	271 (86.3)	633 (92.3)		88 (89.8)	89 (90.8)	
Mechanism of injury						
Acceleration/deceleration	27 (8.7)	68 (10.0)	.003	10 (10.2)	15 (15.3)	.56
Direct impact or fall	252 (81.6)	582 (85.7)		82 (83.7)	77 (78.6)	
Penetrating	30 (9.7)	29 (4.3)		6 (6.1)	6 (6.1)	
Likelihood injury due to abuse						
No concern	244 (77.7)	578 (84.3)	.08	75 (76.5)	77 (78.6)	.94
Possible	17 (5.4)	30 (4.4)		5 (5.1)	6 (6.1)	
Probable	27 (8.6)	41 (6.0)		10 (10.2)	8 (8.2)	
Definite	26 (8.3)	37 (5.4)		8 (8.2)	7 (7.1)	
Glasgow Coma Scale score, mean (SD)	4.83 (1.76)	5.32 (1.84)	<.001	4.88 (1.82)	4.91 (1.75)	.90
Injury severity score, mean (SD)^d^	27.1 (11.6)	26.8 (11.7)	.71	26.4 (10.9)	30.2 (13.1)	.03
Time between injury and monitor placement, median (IQR), h	5.25 (3.75-8.20)	6.98 (4.62-11.5)	<.001	6.23 (3.83-9.13)	5.92 (4.33-11.0)	<.001
Cardiac arrest	32 (10.2)	49 (7.1)	.10	12 (12.2)	11 (11.2)	.82
Pediatric Risk of Mortality score, III, mean (SD)	17.8 (9.86)	16.7 (8.70)	.09	18.3 (9.72)	16.1 (9.29)	.11
Pupil size, mean (SD), mm						
Left	3.47 (1.62)	3.16 (1.44)	.004	3.54 (1.76)	3.08 (1.41)	.05
Right	3.54 (1.64)	3.19 (1.37)	.001	3.42 (1.59)	3.29 (1.44)	.57
Fixed pupils						
Both	84 (26.8)	120 (17.5)	.006	22 (22.4)	19 (19.4)	.39
Either	29 (9.2)	66 (9.6)		10 (10.2)	10 (10.2)	
Neither	183 (58.3)	443 (64.6)		64 (65.3)	62 (63.3)	
Unable to assess or unknown	18 (5.7)	57 (8.3)		2 (2.0)	7 (7.1)	
Partial brain tissue oxygen	24 (7.6)	61 (9.0)	<.001	2 (2.2)	3 (3.2)	.65
Decompressive craniectomy	84 (26.8)	110 (16.2)	<.001	19 (19.4)	18 (18.4)	.86
CT scan results						
Skull fracture	195 (63.5)	425 (64.5)	.77	60 (61.9)	52 (53.6)	.24
Extra-axial hematoma	246 (81.5)	469 (73.2)	.005	77 (80.2)	72 (75.0)	.39
Epidural hematoma	29 (9.4)	61 (9.3)	.93	8 (8.2)	6 (6.2)	.58
Subdural hematoma	225 (73.3)	431 (65.3)	.01	68 (70.1)	69 (71.1)	.87
Hemorrhage						
Intracerebral	177 (57.7)	406 (61.6)	.24	55 (56.7)	48 (49.5)	.31
Intraventricular	98 (31.9)	142 (21.5)	<.001	27 (27.8)	23 (23.7)	.51
Subarachnoid	176 (57.3)	318 (48.3)	.008	60 (61.9)	54 (55.7)	.38
Midline shift supratentorial	125 (40.7)	218 (33.1)	.02	36 (37.1)	40 (41.2)	.56
Contusion	152 (49.5)	340 (51.6)	.55	40 (41.2)	42 (43.3)	.77
Penetrating injury	40 (13.0)	64 (9.7)	.12	11 (11.3)	9 (9.3)	.64
Study hospital						
Free-standing children's hospital	256 (81.5)	446 (65.0)	<.001	83 (84.7)	84 (85.7)	.84
Uses electronic medical records	304 (96.8)	576 (84.0)	<.001	88 (89.8)	96 (98.0)	.01

Abbreviations: CSF, cerebrospinal fluid; CT, computed tomography.

^a^ Matching was conducted using propensity score. Adjustments were made for Hispanic ethnicity, pupil size, PbO2 monitor placement, decompressive craniectomy, extra-axial hematoma, intracranial hemorrhage, free-standing children’s hospital, electronic health record, and site.

^b^ Includes Asian, Native Hawaiian or Pacific Islander, American Indian, and Alaska Native or Inuit individuals and those whose race and ethnicity were unknown by the research team.

^c^ Sites outside the US did not collect this information.

^d^ Calculated internally as the sum of the 3 highest squared Abbreviated Injury Scale body region scores.

**Table 2.** Bivariate Models of Primary and Secondary Outcomes, All Patients

Outcome	Estimate (95% CI)^a^			
	Unadjusted	*P* value	Adjusted^b^	*P* value
Glasgow Outcome Scale–Extended, Pediatric Version^c^^,^^d^	1.36 (0.95-1.96)	.09	1.18 (0.71-1.95)	.53
Death^d^	1.26 (0.85-1.86)	.25	0.74 (0.42-1.31)	.31
Time to death^e^	1.22 (0.86-1.73)	.26	0.78 (0.46-1.32)	.35
Complications^d^				
Respiratory	1.08 (0.72-1.62)	.70	0.84 (0.52-1.36)	.47
Cardiovascular	1.31 (0.79-2.19)	.29	1.05 (0.53-2.07)	.88
General	1.39 (0.90-2.13)	.13	0.97 (0.55-1.72)	.92
Neurological	1.24 (0.89-1.72)	.21	1.15 (0.75-1.76)	.52

^a^ Included 769 participants for models of Glasgow Outcome Scale–Extended, Pediatric Version, 998 participants for survival models, and 997 participants for models of complications.

^b^ Inverse probability of treatment weighting after adjusting for the following remaining imbalances: Hispanic ethnicity, left and right pupil size, whether a partial pressure of blood oxygen monitor was placed, whether a decompressive craniectomy for refractory intracranial pressure was performed, the presence or absence of an extra-axial hematoma, the presence or absence of an intraventricular hemorrhage, whether the study hospital was a free-standing children’s hospital, whether the study hospital used electronic medical records, and study site (when its inclusion did not result in a quasicomplete separation of data points).

^c^ Higher scores indicate worse outcome.

^d^ Expressed as odds ratios. Odds ratios greater than 1 indicate greater odds of outcome for the CSF diversion group compared with the no CSF diversion group.

^e^ Expressed as hazard ratios. Hazard ratios greater than 1 indicate greater risk of outcome for the CSF diversion group compared with the no CSF diversion group per unit of time (days).

**Figure 2.**
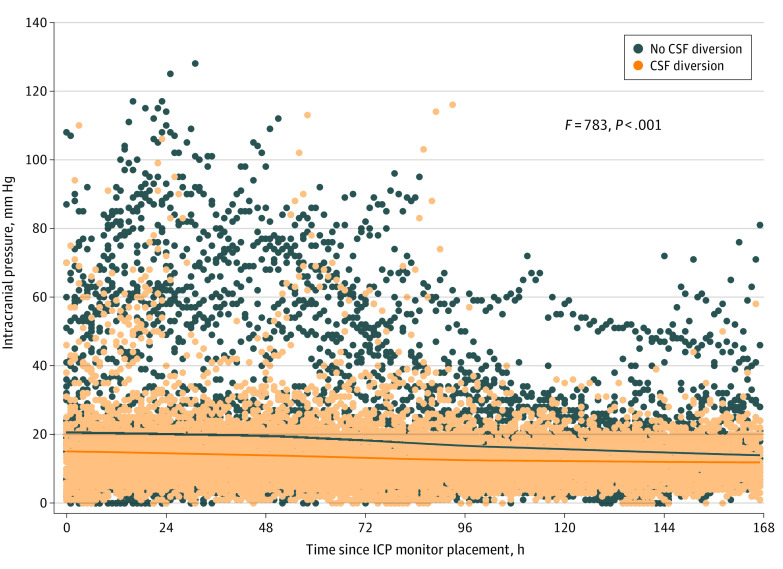
Intracranial Pressure (ICP) Response Between Groups Dots indicate individual data points; lines, trends. CSF indicates cerebrospinal fluid.

In the propensity-matched analysis, there was no difference in the primary outcome between the groups (median [IQR] difference in GOS-EP score, 0 [−2 to 3]) (Table 3). The lack of a difference persisted after controlling for characteristics not balanced through matching (parameter estimate, 0.8574 [95% CI, −1.2165 to 2.9312]; *P* = .96). There was no difference for death at 28 days (OR, 1.18 [95% CI, 0.62-2.52]; *P* = .46) or time to death (hazard ratio [HR], 1.15 [95% CI, 0.66-2.02]; *P* = .61) in adjusted analysis or after adjusting for characteristics not balanced after propensity matching (race, Hispanic ethnicity, injury severity score, time to ICP monitor placement) (28-day mortality: OR, 0.50 [95% CI, 0.21-1.18]; *P* = .11; time to death: HR, 0.55 [95% CI, 0.26-1.16]; *P* = .12) (Table 3). Kaplan-Meier estimates of the time to death were not significantly different between groups (eFigure 1 in Supplement 1). A total of 62 children received delayed CSF diversion, defined as CSF diversion occurring after the initial ICP monitor was placed. A sensitivity analysis including these children in the CSF diversion group did not demonstrate differences between groups (median [IQR] difference in GOS-EP score, 0 [−2 to 3]; *P* = .09) (eFigure 2 and eTable 4 in Supplement 1). In the second sensitivity analysis, a classification tree identified 2 subgroups unlikely to receive the intervention: participants from non-US sites (21 of 295 participants [7.1%] had CSF diversion), and participants from a US site with a mean annual enrollment of fewer than 25 participants and an Abbreviated Injury Scale abdomen score greater than 0 (4 of 39 participants [10.3%] had CSF diversion). This resulted in 148 matched pairs. Outcomes were not different between matched pairs (median [IQR] difference in GOS-EP, 0 [−2 to 3]; *P* = .95) (eFigure 3 in Supplement 1). Using 21 000 hourly ICP readings for the matched cohort, the mean (SD) difference between groups was 4.89 (12.79) mm Hg (95% CI, 4.53-5.24 mm Hg; *P* < .001) (Figure 2) over the study period, with the ICP was lower in the CSF diversion group compared with the no CSF diversion group.

**Table 3.** Models of Primary and Secondary Outcomes, Matched Patients

Outcome	Matched by Propensity score (N = 98 pairs)			
	Estimate	*P* value	Adjusted estimate^a^	
Glasgow Outcome Scale–Extended, Pediatric Version score, median (IQR)	0 (−2 to 3)	.40^b^	NA	NA
Death, OR (95% CI)^c^	1.18 (0.62 to 2.25)	.62	0.50 (0.21 to 1.18)	.11
Time to death, hazard ratio (95% CI) per 1-d increase^c^	1.15 (0.66 to 2.02)	.61	0.55 (0.26 to 1.16)	.12
Complications, OR (95% CI)^c^				
Respiratory	0.90 (0.49 to 1.68)	.75	0.93 (0.42 to 2.04)	.86
Cardiovascular	2.35 (0.78 to 7.04)	.13	2.14 (0.60 to 7.66)	.24
General	1.08 (0.50 to 2.33)	.84	1.1 (0.39 to 3.04)	.86
Neurological	1.09 (0.61 to 1.94)	.77	1.26 (0.62 to 2.58)	.52

Abbreviations: NA, not applicable; OR, odds ratio.

^a^ Including primary race, Hispanic ethnicity, injury severity score, and time between injury and intracranial pressure monitor placement.

^b^ Wilcoxon signed-rank test.

^c^ Estimate greater than 1 indicates greater risk of outcome for the CSF diversion group compared with the no CSF diversion group.

## Discussion

In this comparative effectiveness study, the most comprehensive analysis of children with severe TBI to our knowledge, there was no association of CSF diversion with improved overall outcomes at 6 months after injury. However, in an exploratory analysis, we found an association between CSF diversion and lower ICP. These findings may inform future recommendations for CSF diversion for children with severe TBI.

CSF diversion has been used in children with severe TBI for decades, yet the recommendations supporting its utility are based on limited studies. Shapiro and Marmarou^32^ published a case series of 22 children with CSF diversion, reporting 22% mortality. However, the intent of this early report was to demonstrate the utility of the pressure-volume index, rather than evaluating the effectiveness of CSF diversion. A 2008 study in 23 children by Jagannathan and colleagues^33^ reported 13% mortality and observed that most survivors achieved ICP control. A 2011 case series by Andrade and colleagues^34^ reported outcomes of 11 children with continuous CSF diversion, but comparisons between CSF diversion strategies were not performed.

CSF diversion is also recommended for adults with severe TBI, with recommendations that continuous CSF diversion may be considered to lower ICP burden and CSF diversion may be considered within the first 12 hours for patients with GCS scores less than 6 to lower ICP. The studies supporting these recommendations have similar limitations to those that inform the pediatric guidelines. A study by Nwachuku and colleagues^35^ compared the effectiveness of continuous vs intermittent CSF diversion in 62 participants (31 participants per group) and found that the continuous CSF diversion group had improved ICP (5.66 mm Hg lower than the intermittent group) and fewer episodes of intracranial hypertension. However, a comparison between diversion groups was not performed. A study by Griesdale and colleagues^36^ compared 93 adults with CSF diversion vs 73 adults without this therapy in an uncontrolled observational study. CSF diversion was associated with increased mortality; however, subgroup analysis of patients with GCS scores less than 6 showed some benefit, leading to one of the aforementioned recommendations stated.^12^ A secondary analysis from 2019 by Bales et al^37^ assessed outcomes in the citicoline brain injury treatment trial based on CSF diversion utilization. CSF diversion was performed at the discretion of the site, and a comparison between participants who did and did not receive CSF diversion (224 and 123 participants, respectively) was performed. Bales et al^37^ found that CSF diversion was associated with higher mortality and worse neuropsychological outcomes. The findings of our exploratory analysis related to ICP, which was able to control for covariates and match patients based on relevant characteristics, suggest that CSF diversion could be useful to control intracranial hypertension early after severe TBI in children, as some of these smaller adult studies have indicated.

Our study represents the highest quality of evidence regarding CSF diversion after TBI to out knowledge. Although our study design could only detect associations between the therapy and outcomes and could not discern cause and effect relationships, this method may be the most feasible way to test the effectiveness of this therapy that is already part of contemporary practice. A randomized clinical trial would be a superior method to determine the effectiveness of CSF diversion, if such a study could be accomplished. However, several factors could hamper such a study. Since we did not demonstrate a beneficial association of CSF diversion with overall outcome, estimating an effect size to plan a randomized trial would be challenging. Equipoise regarding CSF diversion may also be negatively impacted by our primary finding related to overall outcome, but our exploratory analysis related to ICP may support such an approach.

### Strengths and Limitations

This study has some strengths as well as limitations. As the largest prospectively enrolled study in this population to our knowledge, we were able to adjust for many measured covariates that might affect outcomes, including more than 1200 unique data elements. However, unmeasured covariates could influence our findings. Our enrollment procedures allowed us to minimize selection bias by including consecutive participants, but these methods limited our ability to obtain outcomes from all participants. This limitation was mitigated to some degree by our follow-up procedures. Our broad inclusion criteria and consecutive enrollment procedures intentionally included children who are often not included in clinical trials, particularly those with penetrating injuries and with abusive head trauma. While this limits the direct comparison of our study with some others, we thought it was important to determine the overall effectiveness of CSF diversion in the entire pediatric TBI population. Despite our large sample size, we were unable to stratify participants into subgroups (GCS scores, sex, age) that might have demonstrated some benefit. While we were able to perform sensitivity analyses, we were unable to evaluate if children with posttraumatic hydrocephalus would benefit. Additionally, while the study included 1000 participants, the participant characteristics and statistical methods yielded a limited number of participants for the primary analysis, which may limit the generalizability of the results.

## Conclusions

This comparative effectiveness study did not find a beneficial association of CSF diversion with GOS-EP at 6 months after severe TBI in children. Further studies will be necessary to determine if there are subsets of children who might benefit. However, in light of our findings, recommendations for CSF diversion may need to be reconsidered.

## Notes

Supplement 1. **eTable 1.** ADAPT Site List **eTable 2.** Data Collection Forms **eTable 3.** Complication Definitions **eFigure 1.** Kaplan-Meier Time to Death **eTable 4.** Sensitivity Analysis for Delayed CSF Diversion **eFigure 2.** Consort Diagram for Sensitivity Analysis **eFigure 3.** Classification Tree and Boxplots of Propensity Scores to Exclude Subgroups Unlikely to Receive CSF Diversion for Sensitivity Analysis

Supplement 2.
The Approaches and Decisions for Acute Pediatric TBI (ADAPT) Investigators

## References

[1] Kompanje EJ, Delwel EJ. The first description of a device for repeated external ventricular drainage in the treatment of congenital hydrocephalus, invented in 1744 by Claude-Nicolas Le Cat. Pediatr Neurosurg. 2003;39(1):10-13. doi:10.1159/000070872 12784070

[2] Srinivasan VM, O’Neill BR, Jho D, Whiting DM, Oh MY. The history of external ventricular drainage. J Neurosurg. 2014;120(1):228-236. doi:10.3171/2013.6.JNS121577 23889138

[3] Sekula RF, Cohen DB, Patek PM, Jannetta PJ, Oh MY. Epidemiology of ventriculostomy in the United States from 1997 to 2001. Br J Neurosurg. 2008;22(2):213-218. doi:10.1080/02688690701832084 18348016

[4] Cushing H. The Third Circulation in Studies of Intracranial Physiology and Surgery. Oxford University Press; 1926.

[5] Becker DP, Miller JD, Ward JD, Greenberg RP, Young HF, Sakalas R. The outcome from severe head injury with early diagnosis and intensive management. J Neurosurg. 1977;47(4):491-502. doi:10.3171/jns.1977.47.4.0491 903803

[6] Marshall LF, Smith RW, Shapiro HM. The outcome with aggressive treatment in severe head injuries—part I: the significance of intracranial pressure monitoring. J Neurosurg. 1979;50(1):20-25. doi:10.3171/jns.1979.50.1.0020 758374

[7] Miller JD, Becker DP, Ward JD, Sullivan HG, Adams WE, Rosner MJ. Significance of intracranial hypertension in severe head injury. J Neurosurg. 1977;47(4):503-516. doi:10.3171/jns.1977.47.4.0503 903804

[8] Narayan RK, Kishore PR, Becker DP, . Intracranial pressure: to monitor or not to monitor: a review of our experience with severe head injury. J Neurosurg. 1982;56(5):650-659. doi:10.3171/jns.1982.56.5.0650 7069477

[9] Bullock R, Chesnut RM, Clifton G, ; Brain Trauma Foundation. Guidelines for the management of severe head injury. Eur J Emerg Med. 1996;3(2):109-127. doi:10.1097/00063110-199606000-00010 9028756

[10] The Brain Trauma Foundation; The American Association of Neurological Surgeons; The Joint Section on Neurotrauma and Critical Care. Guidelines for the management of severe traumatic brain injury. J Neurotrauma. 2000;17(6-7; theme issue):449-554.

[11] Bratton SL, Chestnut RM, Ghajar J, ; Brain Trauma Foundation; American Association of Neurological Surgeons; Congress of Neurological Surgeons; Joint Section on Neurotrauma and Critical Care; AANS/CNS. Guidelines for the management of severe traumatic brain injury: VII—intracranial pressure monitoring technology. J Neurotrauma. 2007;24(suppl 1):S45-S54. doi:10.1089/neu.2007.9989 17511545 10.1089/neu.2007.9989

[12] Carney N, Totten AM, O’Reilly C, . Guidelines for the management of severe traumatic brain injury, fourth edition. Neurosurgery. 2017;80(1):6-15. doi:10.1227/NEU.0000000000001432 27654000

[13] Adelson PD, Bratton SL, Carney NA, ; American Association for Surgery of Trauma; Child Neurology Society; International Society for Pediatric Neurosurgery; International Trauma Anesthesia and Critical Care Society; Society of Critical Care Medicine; World Federation of Pediatric Intensive and Critical Care Societies. Guidelines for the acute medical management of severe traumatic brain injury in infants, children, and adolescents: chapter 10—the role of cerebrospinal fluid drainage in the treatment of severe pediatric traumatic brain injury. Pediatr Crit Care Med. 2003;4(3)(suppl):S38-S39. 12847346

[14] Kochanek PM, Carney N, Adelson PD, ; American Academy of Pediatrics-Section on Neurological Surgery; American Association of Neurological Surgeons/Congress of Neurological Surgeons; Child Neurology Society; European Society of Pediatric and Neonatal Intensive Care; Neurocritical Care Society; Pediatric Neurocritical Care Research Group; Society of Critical Care Medicine; Paediatric Intensive Care Society UK; Society for Neuroscience in Anesthesiology and Critical Care; World Federation of Pediatric Intensive and Critical Care Societies. Guidelines for the acute medical management of severe traumatic brain injury in infants, children, and adolescents—second edition. Pediatr Crit Care Med. 2012;13(suppl 1):S1-S82. doi:10.1097/PCC.0b013e318259ee85 22217782

[15] Kochanek PM, Tasker RC, Carney N, . Guidelines for the management of pediatric severe traumatic brain injury, third edition: update of the Brain Trauma Foundation guidelines. Pediatr Crit Care Med. 2019;20(3S)(suppl 1):S1-S82. doi:10.1097/PCC.0000000000001735 30829890

[16] Kochanek PM, Tasker RC, Bell MJ, . Management of pediatric severe traumatic brain injury: 2019 consensus and guidelines-based algorithm for first and second tier therapies. Pediatr Crit Care Med. 2019;20(3):269-279. doi:10.1097/PCC.0000000000001737 30830015

[17] Bell MJ, Adelson PD, Hutchison JS, ; Multiple Medical Therapies for Pediatric Traumatic Brain Injury Workgroup. Differences in medical therapy goals for children with severe traumatic brain injury-an international study. Pediatr Crit Care Med. 2013;14(8):811-818. doi:10.1097/PCC.0b013e3182975e2f 23863819 PMC4455880

[18] Miller Ferguson N, Sarnaik A, Miles D, ; Investigators of the Approaches and Decisions in Acute Pediatric Traumatic Brain Injury (ADAPT) Trial. Abusive Head trauma and mortality—an analysis from an international comparative effectiveness study of children with severe traumatic brain injury. Crit Care Med. 2017;45(8):1398-1407. doi:10.1097/CCM.0000000000002378 28430697 PMC5511066

[19] Murphy S, Thomas NJ, Gertz SJ, ; Investigators of the Approaches and Decisions in Acute Pediatric Traumatic Brain Injury (ADAPT) Study. Tripartite stratification of the Glasgow Coma Scale in children with severe traumatic brain injury and mortality: an analysis from a multi-center comparative effectiveness study. J Neurotrauma. 2017;34(14):2220-2229. doi:10.1089/neu.2016.4793 28052716 PMC5510706

[20] Sarnaik A, Ferguson NM, O’Meara AMI, ; Investigators of the ADAPT Trial. Age and mortality in pediatric severe traumatic brain injury: results from an international study. Neurocrit Care. 2018;28(3):302-313. doi:10.1007/s12028-017-0480-x 29476389 PMC10655613

[21] Adelson PD, Pineda J, Bell MJ, ; Pediatric TBI Demographics and Clinical Assessment Working Group. Common data elements for pediatric traumatic brain injury: recommendations from the working group on demographics and clinical assessment. J Neurotrauma. 2012;29(4):639-653. doi:10.1089/neu.2011.1952 21939389 PMC3289844

[22] Duhaime AC, Holshouser B, Hunter JV, Tong K. Common data elements for neuroimaging of traumatic brain injury: pediatric considerations. J Neurotrauma. 2012;29(4):629-633. doi:10.1089/neu.2011.1927 21671798 PMC3289846

[23] McCauley SR, Wilde EA, Anderson VA, ; Pediatric TBI Outcomes Workgroup. Recommendations for the use of common outcome measures in pediatric traumatic brain injury research. J Neurotrauma. 2012;29(4):678-705. doi:10.1089/neu.2011.1838 21644810 PMC3289848

[24] Beers SR, Wisniewski SR, Garcia-Filion P, . Validity of a pediatric version of the Glasgow Outcome Scale–Extended. J Neurotrauma. 2012;29(6):1126-1139. doi:10.1089/neu.2011.2272 22220819 PMC3325553

[25] McCaffrey DF, Griffin BA, Almirall D, Slaughter ME, Ramchand R, Burgette LF. A tutorial on propensity score estimation for multiple treatments using generalized boosted models. Stat Med. 2013;32(19):3388-3414. doi:10.1002/sim.5753 23508673 PMC3710547

[26] McCaffrey DF, Ridgeway G, Morral AR. Propensity score estimation with boosted regression for evaluating causal effects in observational studies. Psychol Methods. 2004;9(4):403-425. doi:10.1037/1082-989X.9.4.403 15598095

[27] RAND Corporation. Downloads. Accessed June 6, 2022. https://www.rand.org/statistics/twang/downloads.html

[28] Austin PC. Propensity-score matching in the cardiovascular surgery literature from 2004 to 2006: a systematic review and suggestions for improvement. J Thorac Cardiovasc Surg. 2007;134(5):1128-1135. doi:10.1016/j.jtcvs.2007.07.021 17976439

[29] Austin PC. A critical appraisal of propensity-score matching in the medical literature between 1996 and 2003. Stat Med. 2008;27(12):2037-2049. doi:10.1002/sim.3150 18038446

[30] Allison PD. Fixed Effects Regression Methods for Longitudinal Data Using SAS. SAS Institute; 2005.

[31] Bang H, Robins JM. Doubly robust estimation in missing data and causal inference models. Biometrics. 2005;61(4):962-973. doi:10.1111/j.1541-0420.2005.00377.x 16401269

[32] Shapiro K, Marmarou A. Clinical applications of the pressure-volume index in treatment of pediatric head injuries. J Neurosurg. 1982;56(6):819-825. doi:10.3171/jns.1982.56.6.0819 7077382

[33] Jagannathan J, Okonkwo DO, Yeoh HK, . Long-term outcomes and prognostic factors in pediatric patients with severe traumatic brain injury and elevated intracranial pressure. J Neurosurg Pediatr. 2008;2(4):240-249. doi:10.3171/PED.2008.2.10.240 18831656

[34] Andrade AF, Paiva WS, Amorim RL, . Continuous ventricular cerebrospinal fluid drainage with intracranial pressure monitoring for management of posttraumatic diffuse brain swelling. Arq Neuropsiquiatr. 2011;69(1):79-84. doi:10.1590/S0004-282X2011000100016 21359428

[35] Nwachuku EL, Puccio AM, Fetzick A, . Intermittent versus continuous cerebrospinal fluid drainage management in adult severe traumatic brain injury: assessment of intracranial pressure burden. Neurocrit Care. 2014;20(1):49-53. doi:10.1007/s12028-013-9885-3 23943318

[36] Griesdale DE, McEwen J, Kurth T, Chittock DR. External ventricular drains and mortality in patients with severe traumatic brain injury. Can J Neurol Sci. 2010;37(1):43-48. doi:10.1017/S031716710000963X 20169772

[37] Bales JW, Bonow RH, Buckley RT, Barber J, Temkin N, Chesnut RM. Primary external ventricular drainage catheter versus intraparenchymal ICP monitoring: outcome analysis. Neurocrit Care. 2019;31(1):11-21. doi:10.1007/s12028-019-00712-9 31037639

