# Errors in Abstract and Main Text Results

**DOI:** 10.1001/jamanetworkopen.2022.32865

**Published:** 2022-08-31

In the Original Investigation titled “Comparative Effectiveness of Diversion of Cerebrospinal Fluid for Children With Severe Traumatic Brain Injury,”^1^ published July 8, 2022, there were errors in the Results sections of the Abstract and the main text. In the Abstract, the median (IQR) Glasgow Outcome Score–Extended for Pediatrics should have been 0 (−2 to 3) (*P* = .40) and the mean (SD) difference in intracranial pressure should have been 3.98 (0.14) mm HG. In the main text, the *P* value for the propensity-matched analysis of odds ratio of death at 28 days should have been *P* = .62. This article has been corrected.^1^
