# Supplementary material for: Comparative Effectiveness of Diversion of Cerebrospinal Fluid for Children With Severe Traumatic Brain Injury
Source: JAMA Netw Open. 2022 Jul 8;5(7):e2220969. doi: 10.1001/jamanetworkopen.2022.20969 (PMC9270700; doi:10.1001/jamanetworkopen.2022.20969)
Supplement: Supplement 1. — eTable 1. ADAPT Site List eTable 2. Data Collection Forms eTable 3. Complication Definitions eFigure 1. Kaplan-Meier Time to Death eTable 4. Sensitivity Analysis for Delayed CSF Diversion eFigure 2. Consort Diagram for Sensitivity Analysis eFigure 3. Classification Tree and Boxplots of Propensity Scores to Exclude Subgroups Unlikely to Receive CSF Diversion for Sensitivity Analysis [file jamanetwopen-e2220969-s001.pdf]

Supplemental Online Content

Bell MJ, Rosario BL, Kochanek PM, et al; Approaches and Decisions for Acute Pediatric TBI (ADAPT) Investigators. Comparative effectiveness of diversion of cerebrospinal fluid for children with severe traumatic brain injury. *JAMA Netw Open*. 2022;5(7):e2220969. doi:10.1001/jamanetworkopen.2022.20969

eTable 1. ADAPT Site List

eTable 2. Data Collection Forms

eTable 3. Complication Definitions

eFigure 1. Kaplan-Meier Time to Death

eTable 4. Sensitivity Analysis for Delayed CSF Diversion

eFigure 2. Consort Diagram for Sensitivity Analysis

eFigure 3. Classification Tree and Boxplots of Propensity Scores to Exclude Subgroups Unlikely to Receive CSF Diversion for Sensitivity Analysis

This supplemental material has been provided by the authors to give readers additional information about their work.

© 2022 Bell MJ et al. *JAMA Network Open.*

eTable 1. ADAPT Site List

**ADAPT Site List**
Children's Healthcare of Atlanta, Atlanta GA
Children's National Medical Center, Washington, DC
Children's Hospital of Michigan, Detroit, MI
Columbia University, New York, NY
Boston Children's Hospital, Boston, MA
Johns Hopkins University, Baltimore, MD
Carolinas Medical Center Levine Children's Hospital, Charlotte NC
Massachusetts General Hospital, Boston MA
Miami Children's Hospital, Miami FL
Lurie Children's Hospital, Chicago IL
Nationwide Children's Hospital, Columbus OH
Phoenix Children's Hospital, Phoenix AZ
Penn State University - Hershey, Hershey PA
Texas Children's Hospital, Houston TX
The Reagents of University California Davis, Sacramento, CA
University of Alabama-Birmingham, Birmingham AL
University of California, Los Angeles, Los Angeles, CA
University of California, San Diego, San Diego CA
Cincinnati Children's Hospital Medical Center, Cincinnati OH
Vanderbilt University, Nashville TN
University of Michigan Reagents, Ann Arbor, MI
Children's Hospital of Pennsylvania, Philadelphia PA
University of Pittsburgh, Pittsburgh PA
Children's Hospital of Los Angeles, Los Angeles CA
Le Bonheur Children's Hospital, Memphis TN
University of Nebraska Medical Center, Omaha NE
University of Texas Southwestern, Dallas TX
University of Utah, Salt Lake City UT
University of Washington, Seattle WA
Washington University of St. Louis, St Louis MO
University of Wisconsin - Madison WI
University of Colorado, Aurora CO
Children's Hospital of Richmond, Richmond VA
Hackensack University Medical Center, Hackensack NJ
University of Iowa, Iowa City IA
Hospital Vall d' Hebron, Barcelona, Spain
Erasmus Medical Center, Rotterdam Netherlands
Newcastle upon Tyne Hospital, Newcastle, UK
North Bristol NHS Trust, UK
Royal Manchester Children's Hospital, Manchester, UK
Alder Hey Children's NHS Foundation Trust Liverpool, UK
Addenbrookes Hospital, Cambridge, UK

© 2022 Bell MJ et al. *JAMA Network Open.*

Kings College Hospital, London, UK
Leeds Teaching Hospital NHS Trust, Leeds UK
University Hospital Southampton, Southampton UK
Great Ormond Street, London UK
Birmingham Children's Hospital, Birmingham UK
Starship Children's Hospital, Auckland NZ
Children's Health Queensland Hospital and Health Service, Brisbane Australia
Children's Research Institute, Royal Children's Hospital, Melbourne Australia
Perth Children's Hospital, Perth Australia
All India Institute of Medical Sciences, New Delhi India
Red Cross War Memorial Children's Hospital, Cape Town, South Africa

© 2022 Bell MJ et al. *JAMA Network Open.*

eTable 2. Data Collection Forms

ADAPT

Study ID: [-]

Site: [ ]

Date of ICP Monitor placement: _ _ /_ _ /_ _ _ _ **Time:** ___________ *(use 24-hour clock)*

(at Study Hospital)

Time point: **Acute Hospitalization**

Packet Type: **Acute**

Summary of Assessments to be completed:

Collected Once:

DEMOG	Demographics
AIS	Abbreviated Injury Score
PREH	Pre-Hospital Complications
RESUS	Resuscitation Form
PRISM	PRISM III
SURG	Surgeries & Procedures for ICP (at least one is expected)
SCANS	Radiology Transmission Form

*Collected Daily:**

FLDDY	Fluids
MEDIC	Medications
NEURX	Neurological Exam (Qualifying Exam, ICU Days 1 – 7, ICU Discharge, Hospital Discharge)
PILOT	PILOT Therapies

*Collected Hourly:**

PHYSO	Physiology
LABS	Labs
NUTR	Nutrition Labs
NUTR2	Nutritional Support

© 2022 Bell MJ et al. *JAMA Network Open.*

GAS	Blood Gases
MEDS	Hourly Meds

Completed at hospital discharge:

DISCH	Hospital Discharge
COMPLIC	Medical & Surgical Complications of TBI

*Because the length of the ICU stay will vary, this Forms Packet has been generated with one copy of each data collection sheet. For data collected on multiple days, additional copies of individual data sheets should be printed as needed from the study website.

© 2022 Bell MJ et al. *JAMA Network Open.*

Form Date:_____/_____/___________ Study ID: -

Entered:_____/____/__________Initials:__________ **For office use only.**

ADAPT: Demographics (DEMOG)

Staff ID -------------- ____

1. Gender: ☐ Female ☐ Male

2. a) Race: *(Please select all appropriate categories for subjects of multiracial origin.)*

☐ **White**
If checked, specify below only if known:
☐ North American
☐ South American
☐ European
☐ Middle eastern
☐ North African

☐ **Asian**
If checked, specify below only if known:
☐ South Asian (Indian subcontinent)
☐ Far Eastern Asian

☐ **Black**
If checked, specify below only if known:
☐ African American
☐ African

☐ **Native Hawaiian / Pacific Islander**
If checked, specify below only if known:
☐ Native Hawaiian
☐ Pacific Islander

☐ **Indian**
If checked, specify below only if known:

☐ **Alaska Native / Inuit:**
If checked, specify below only if known:
☐ Alaska Native

☐ **Not Allowed**

☐ **Unknown**

b) Ethnicity: *(Select **only** if White-With no sub-category, White-North American, Black-With no sub-category, or Black-African American)*

☐ Hispanic or Latino
☐ Not Hispanic or Latino

3. Date of Birth:_____/ /__________(mm/dd/yyyy)

4. Height (cm):_______.

5. Weight (kg):_______.

© 2022 Bell MJ et al. *JAMA Network Open.*

6. Head circumference (cm): _______ .

7. Cause of TBI:
   - ☐ Railway accidents
   - ☐ Motor vehicle traffic accident
   - ☐ Motor vehicle nontraffic accidents
   - ☐ Other road vehicle accidents
   - ☐ Water transport accidents
   - ☐ Air and space transport accident
   - ☐ Vehicle accident not elsewhere classified
   - ☐ Accidental falls
   - ☐ Accidents caused by fire and flames
   - ☐ Accidents due to natural & environmental factors
   - ☐ Suicide & self-inflicted injury
   - ☐ Homicide & injury purposely inflicted by other persons
   - ☐ Terrorism
   - ☐ Injury resulting from operations of war
   - ☐ Other accidents If Other, Specify:__________________________

Study ID: -

8. Type of TBI:

Select one: ☐ Closed ☐ Blast

☐ Penetrating ☐ Crush

9. Mechanism of TBI:

Select one: ☐ Acceleration/Deceleration ☐ Ground level fall

☐ Direct impact: blow to head ☐ Fall from height > 1meter (3ft)
☐ Direct impact: head against object ☐ Gunshot wound
☐ Crush ☐ Fragment (incl. shell/shrapnel)
☐ Blast ☐ Other penetrating brain injury

10. Likelihood that injury was due to abusive head trauma?

Select one: ☐ No concern ☐ Possible

☐ Probable ☐ Definite

11. Likelihood that injury was intentional?

Select one: ☐ No concern ☐ Possible

☐ Probable ☐ Definite

12. Likelihood that the injury was self-inflicted?

Select one: ☐ No concern ☐ Possible

☐ Probable ☐ Definite

13. Likelihood that the patient was under the influence of alcohol and/or drugs?

Select one: ☐ None ☐ Suspected

© 2022 Bell MJ et al. *JAMA Network Open.*

☐ Confirmed ☐ Unknown

14. Date of arrival at study hospital: ____ /____/ _________ (mm/dd/yyyy)

15. Time of arrival (use 24-hour clock): _____ : _____

16. Patient transported to study hospital from:

☐ Scene of accident / injury

☐ Home

☐ Other hospital →

If transported from another hospital:

a. Date of arrival at other hospital: _____ /_____/ ___________
mm dd yyyy

b. Time of arrival at other hospital (use 24-hour clock): [illegible] : [illegible]

17. Qualifying GCS prior to placement of ICP monitor:

Eye__________
Verbal_______
Motor_______
Total _______

© 2022 Bell MJ et al. *JAMA Network Open.*

18. Primary Language (select **only** one):

☐ English ☐ Spanish ☐ Sign Language
☐ Chinese ☐ French ☐ German ☐ Other, Specify ___________

19. In order to obtain the population size of the town/ city/ state where the patient lives, please indicate whether the patient has a Zip code or Postal code and indicate that code.

☐ Patient Zip Code ***(If zip code, indicate the zip code)*** Zip Code: ____________
☐ Patient Postal Code ***(If postal code, indicate the postal code)*** Postal Code: ______________

© 2022 Bell MJ et al. *JAMA Network Open.*

Form Date:_____/_____/ __________ Study ID: -

Entered:_____/_____/_________Initials:___________ **For office use only.**

Staff ID ---------------- _____

ADAPT:
Abbreviated Injury Score (AIS)

**Body Region**	**Highest AIS**
Head	
Face	
Neck	
Thorax	
Abdomen	
Spine	
Upper Extremities	
Lower Extremities	
External	

© 2022 Bell MJ et al. *JAMA Network Open.*

Form Date:_____/_____/ __________ Study ID: -

Entered:_____/____/__________Initials: __________

Staff ID ----------------_____

ADAPT:
Pre-Hospital Events (PREH)

Use this form to document the following events that occurred *after* the patient's injury but*prior to their arrival at the study hospital*. Please document these events if experienced by the patient during transportation to the study hospital as well as at a non-study hospital to which the patient may have been initially transported.

Apnea: ☐ Yes ☐ No ☐ Suspected ☐ Unknown

Aspiration: ☐ Yes ☐ No ☐ Suspected ☐ Unknown

Cardiac Arrest: ☐ Yes ☐ No ☐ Suspected ☐ Unknown

Hypotension: ☐ Yes ☐ No ☐ Suspected ☐ Unknown

Hypoxia: ☐ Yes ☐ No ☐ Suspected ☐ Unknown

Seizure: ☐ Yes ☐ No ☐ Suspected ☐ Unknown

Hyperthermia: ☐ Yes ☐ No ☐ Suspected ☐ Unknown

Hypothermia: ☐ Yes ☐ No ☐ Suspected ☐ Unknown

Hyperventilation: ☐ Yes ☐ No ☐ Suspected ☐ Unknown

© 2022 Bell MJ et al. *JAMA Network Open.*

Form Date:_____/_____/ __________ Study ID: [-]

Entered:_____/____/_________Initials:__________**For office use only.**

Staff ID ----------------_____

ADAPT:
Resuscitation Form (RESUS)

Use this form to document the following information regarding the patient from *time of arrivalto study hospital* to *insertion of ICP monitor*.

COMPLICATIONS

Cardiac Arrest · Yes · No

Hypotension · Yes · No

Hypoxia · Yes · No

Seizure · Yes · No

Hyperthermia · Yes · No

Hypothermia · Yes · No

Hyperventilation · Yes · No

MEDICATIONS

Anticonvulsant · Yes · No

If yes, check all that apply

- Phenytoin
- Fosphenytoin
- Keppra (Levetiracetam)
- Phenobarbital
- Oxcarbazepine (Trileptal)
- Primidone (Mysoline)

Please Specify ________________________

Hypertonic Saline · Yes · No | If 'Yes', % Concentration_______ Calculate the total dose in cc _____________

Mannitol (Osmitrol) · Yes · No | If 'Yes', calculate the total dose in grams _____________

Barbiturates · Yes · No | If 'Yes', calculate the total dose in mg _____________

FLUIDS

Fluids In:

____

Fluids Out: _______

© 2022 Bell MJ et al. *JAMA Network Open.*

LABS

Hgb: ____ .

Platelets: ________

WBC ____ .

Na ______

PT ____ .

PTT ______ .

INR ____ .

PH . ______

PaO2 ______

PCO2 ______

HCO3 ____

Cortisol ____ .

© 2022 Bell MJ et al. *JAMA Network Open.*

Form Date:_____/_____/ __________ Study ID: -

Entered:_____/____/_________Initials:__________ **For office use only.**

Staff ID ---------------- ____

ADAPT: PRISM III (PRISM)

Measurements should be recorded for the *first 12 hours* from 'time of arrival' at study hospital.

1. CARDIO/NEURO/VITAL SIGNS

   Lowest Systolic BP (mmHg): _______

   Highest Heart Rate (beats/min): _______

   Highest Temperature (°C):_____.__

   Lowest Temperature (°C): __ __.__

2. MENTAL STATUS

   Lowest GCS Score: *(Indicate the lowest GCS Score with no pharmacological paralysis)*

   __ __

   For Pupillary Reflexes, indicate type:

   ☐ Both Reactive ☐ One Fixed, One Reactive ☐ Both Fixed (fixed must be >3mm)

3. ACID-BASE/BLOOD GASES *(Check box if collected and record the amount)*

   ☐ Lowest pH (mmol/L): __. _____

   ☐ Lowest Total CO_2 (mmol/L): __ ___. _____

   ☐ Highest pH: __. _____

   ☐ Highest pCO_2 (mmHg): _______

   ☐ Lowest PaO_2 (mmHg): _______

   ☐ Highest Total CO_2 (mmol/L) _____

4. CHEMISTRY TESTS *(Check box if collected and record the amount)*

   ☐ Highest Glucose (mg/dL): _______

   ☐ Highest Potassium (mmol/L):___.

   ☐ Highest Blood Urea Nitrogen (mg/dL): _______

   ☐ Highest Creatinine (mg/dL):_____.

© 2022 Bell MJ et al. *JAMA Network Open.*

Study ID: ___ - ___

5. HEMATOLOGY *(Check box if collected and record the amount)*

☐ Lowest WBC (x10^3/µL):_____. _____

☐ Lowest Platelets (x10^3/µL): _________

☐ Highest PT (in seconds):_____.

☐ Highest PTT (in seconds):_______.

© 2022 Bell MJ et al. *JAMA Network Open.*

Form Date:_____/_____/ __________ Study ID: [-]

Entered:_____/____/_________Initials:_______**For office use only.**

Staff ID ----------------

ADAPT:
Surgeries & Procedures for ICP (SURG)

Complete one form per event(s).

Surgery Date:______/_____/__________ Surgery Time______:______(24-hour clock)

☐ Yes ☐ No **Intracranial monitor placement?**

a) What type of monitor(s) were placed *during this surgery*? (check all that apply)

☐ Yes ☐ No **EVD**

Type of drainage:

☐ Continuous ☐ Intermittent ☐ None

What position was EVD zeroed at placement?

☐ Head ☐ Ear ☐ Chest

☐ Yes ☐ No **Intraparenchymal catheter**

If "Yes" to both EVD and Intraparenchymal monitor placement, was a single device placed to accomplish both things?

☐ Yes ☐ No

Is the ICP reading reported from each probe clearly documented?

☐ Yes ☐ No **PBO2**

☐ Yes ☐ No **Other monitor, specify:** ________________________________

b) Is this the initial ICP monitor placed, qualifying the child for ADAPT enrollment?

☐ Yes ☐ No

Was this initial ICP monitor placed more than 24 hours after injury?

☐ Yes ☐ No

Reason for delay:

☐ Yes ☐ No **Subject had near normal GCS (GCS 13-15), then deteriorated.**
☐ Yes ☐ No **Subject had a moderate GCS (GCS 9-11), then deteriorated.**
☐ Yes ☐ No **Clinical team believed subject would improve but failed.**
☐ Yes ☐ No **Other resuscitative/surgical procedures precluded ICP monitoring.**
☐ Yes ☐ No **Transfer to site hospital caused delay in monitor placement.**

© 2022 Bell MJ et al. *JAMA Network Open.*

Form Date: / / Study ID: -

☐ Yes ☐ No **Evacuation of lesion?**

Check all that apply:

☐ Yes ☐ No **Epidural**

☐ Yes ☐ No **Subdural**

☐ Yes ☐ No **Intraparenchymal**

☐ Yes ☐ No **Decompressive craniectomy for refractory ICP?**

Specify (select one):

☐ **Bilateral** ☐ **Unilateral**

☐ Yes ☐ No **Removal of Intracranial monitor (less than 7 days after placement)?**

Which monitor was removed? Check all that apply:

☐ Yes ☐ No **EVD**

☐ Yes ☐ No **Intraparenchymal catheter**

☐ Yes ☐ No **PBO2**

☐ Yes ☐ No **Other monitor** → If 'Yes', Specify: ____________________

☐ Yes ☐ No **Other, specify:** ____

ADAPT:

© 2022 Bell MJ et al. *JAMA Network Open.*

Form Date:_____/_____/ __________

Study ID: -

Entered: / / Initials: **For office use only.**

Staff ID ----------------- _____

ADAPT:

ICU Stay – Fluids In/Out (FLDDY)

Fluids are Tabulated once every 24 hours

Timepoint	Day 1	Day 2	Day 3	Day 4	Day 5	Day 6	Day 7
Start Date	____ /____/_________	____ /____/_________	____ /____/_________	____ /____/_________	____ /____/_________	____ /____/_________	____ /____/_________
Start Hour	____ : 00	____ : 00	____ : 00	____ : 00	____ : 00	____ : 00	____ : 00
Fluids In:							
PRBC's (ml)							
Whole Blood (ml)							
Platelets (ml)							
FFP (ml)							
Colloids (ml)							
Sodium In:							
TPN (% Na Concentration) (mEq/kg/day)							
NaCl medication supplements (mEq)							
Total 24-hour Fluids in (ml)							
Fluids Out:							
Urine (ml)							
CSF (ml)							
NG/OG (ml)							
Other Out (ml)							

© 2022 Bell MJ et al. *JAMA Network Open.*

Form date:_____/_____/ __________ Study ID: [-]

Entered:_____/____/_________Initials:__________ **For office use only.**

Staff ID ----------------_____

ADAPT:
ICU Stay – Medications (MEDIC)

Date_____/_____/__________ **PICU Day # ______**

Paralytics · Yes · No

If yes, check all that apply

↓ **<u>If 'checked', was this a continuous infusion?</u>**

· Cisatracurium	· Yes · No
· Vecuronium	· Yes · No
· Pancuronium	· Yes · No
· Rocuronium	· Yes · No
	[illegible]

Please Specify ________________________

Narcotics/Sedation · Yes · No

If yes, check all that apply

↓ **<u>If 'checked', was this a continuous infusion?</u>**

· Fentanyl	· Yes · No
· Morphine	· Yes · No
· Propofol	· Yes · No
· Ativan (Lorazepam)	· Yes · No
· Diazepam (Valium)	· Yes · No
· Demerol (Meperidine)	· Yes · No
· Hydromorphone (Dilaudid)	· Yes · No
	[illegible]

Please Specify ________________________

© 2022 Bell MJ et al. *JAMA Network Open.*

Study ID: -

Anticonvulsant · Yes · No

If yes, check all that apply

- Phenytoin
- Fosphenytoin
- Keppra (Levetiracetam)
- Phenobarbital
- Oxcarbazepine (Trileptal)
- Primidone (Mysoline)

Please Specify ___________________________

Vasoactive · Yes · No

If yes, check all that apply

- Dobutamine
- Dopamine
- Epinephrine
- Isoproterenol
- Labetelol
- Nitroglycerin

Please Specify ___________________________

Steroids · Yes · No

If yes, check all that apply

- Methylprednisolone
- Hydrocortisone

Barbiturates · Yes · No

If yes, check all that apply

- Pentobarbital

Other Meds · Yes · No If

yes, check all that apply

- Furosemide
- Bumex

© 2022 Bell MJ et al. *JAMA Network Open.*

Form Date:_____/_____/ __________ Study ID: -

Entered:_____/_____/__________ Initials: ________ **For office use only.**

Staff ID -------------- _____

ADAPT:
Neurological Exam (NEURX)

	Qualifying Exam	Day 1	Day 2	Day 3	Day 4	Day 5	Day 6	Day 7	ICU Discharge	Hospital Discharge
**Test Date**	___/___/	___/___/ _ _ _ _	___/___/	___/___/ _ _ _ _	___/___/	___/___/ _ _ _ _	___/___/	___/___/ _ _ _ _	___/___/	___/___/ _ _ _ _
**Status** (check all that apply)	☐ Paralyzed ☐ Sedated ☐ Intubated	☐ Paralyzed ☐ Sedated ☐ Intubated	☐ Paralyzed ☐ Sedated ☐ Intubated	☐ Paralyzed ☐ Sedated ☐ Intubated	☐ Paralyzed ☐ Sedated ☐ Intubated	☐ Paralyzed ☐ Sedated ☐ Intubated	☐ Paralyzed ☐ Sedated ☐ Intubated	☐ Paralyzed ☐ Sedated ☐ Intubated		
**GCS:**		***GCS data is not expected if the patient is medically paralyzed.***								
**Eye**	_	_	_	_	_	_	_	_	_	_
**Verbal**	_	_	_	_	_	_	_	_	_	_
**Motor**	_	_	_	_	_	_	_	_	_	_
**Total**	_ _	_ _	_ _	_ _	_ _	_ _	_ _	_ _	_ _	_ _
**Pupil Size: Right**	__ mm	__ mm	__ mm	__ mm	__ mm	__ mm	__ mm	__ mm	__ mm	__ mm
**Left**	__ mm	__ mm	__ mm	__ mm	__ mm	__ mm	__ mm	__ mm	__ mm	__ mm
**Pupil Reaction Right**	☐ Normal ☐ Sluggish ☐ Fixed ☐ Unable to assess	☐ Normal ☐ Sluggish ☐ Fixed ☐ Unable to assess	☐ Normal ☐ Sluggish ☐ Fixed ☐ Unable to assess	☐ Normal ☐ Sluggish ☐ Fixed ☐ Unable to assess	☐ Normal ☐ Sluggish ☐ Fixed ☐ Unable to assess	☐ Normal ☐ Sluggish ☐ Fixed ☐ Unable to assess	☐ Normal ☐ Sluggish ☐ Fixed ☐ Unable to assess	☐ Normal ☐ Sluggish ☐ Fixed ☐ Unable to assess	☐ Normal ☐ Sluggish ☐ Fixed ☐ Unable to assess	☐ Normal ☐ Sluggish ☐ Fixed ☐ Unable to assess
**Left**	☐ Normal ☐ Sluggish ☐ Fixed ☐ Unable to assess	☐ Normal ☐ Sluggish ☐ Fixed ☐ Unable to assess	☐ Normal ☐ Sluggish ☐ Fixed ☐ Unable to assess	☐ Normal ☐ Sluggish ☐ Fixed ☐ Unable to assess	☐ Normal ☐ Sluggish ☐ Fixed ☐ Unable to assess	☐ Normal ☐ Sluggish ☐ Fixed ☐ Unable to assess	☐ Normal ☐ Sluggish ☐ Fixed ☐ Unable to assess	☐ Normal ☐ Sluggish ☐ Fixed ☐ Unable to assess	☐ Normal ☐ Sluggish ☐ Fixed ☐ Unable to assess	☐ Normal ☐ Sluggish ☐ Fixed ☐ Unable to assess

© 2022 Bell MJ et al. *JAMA Network Open.*

Form Date:_____/_____/__________ Study ID: -

	Qualifying Exam	Day 1	Day 2	Day 3	Day 4	Day 5	Day 6	Day 7	ICU Discharge	Hospital Discharge
**Pupil Shape:** Right	☐ Round<br>☐ Oval<br>☐ Unable to assess	☐ Round<br>☐ Oval<br>☐ Unable to assess	☐ Round<br>☐ Oval<br>☐ Unable to assess	☐ Round<br>☐ Oval<br>☐ Unable to assess	☐ Round<br>☐ Oval<br>☐ Unable to assess	☐ Round<br>☐ Oval<br>☐ Unable to assess	☐ Round<br>☐ Oval<br>☐ Unable to assess	☐ Round<br>☐ Oval<br>☐ Unable to assess	☐ Round<br>☐ Oval<br>☐ Unable to assess	☐ Round<br>☐ Oval<br>☐ Unable to assess
Left	☐ Round<br>☐ Oval<br>☐ Unable to assess	☐ Round<br>☐ Oval<br>☐ Unable to assess	☐ Round<br>☐ Oval<br>☐ Unable to assess	☐ Round<br>☐ Oval<br>☐ Unable to assess	☐ Round<br>☐ Oval<br>☐ Unable to assess	☐ Round<br>☐ Oval<br>☐ Unable to assess	☐ Round<br>☐ Oval<br>☐ Unable to assess	☐ Round<br>☐ Oval<br>☐ Unable to assess	☐ Round<br>☐ Oval<br>☐ Unable to assess	☐ Round<br>☐ Oval<br>☐ Unable to assess
	**Note: Gaze, Corneal and Cough/Gag/Swallow data is not expected if the patient is medically paralyzed.**									
**Gaze**	☐ Normal<br>☐ Abnormal<br>☐ Not Tested	☐ Normal<br>☐ Abnormal<br>☐ Not Tested	☐ Normal<br>☐ Abnormal<br>☐ Not Tested	☐ Normal<br>☐ Abnormal<br>☐ Not Tested	☐ Normal<br>☐ Abnormal<br>☐ Not Tested	☐ Normal<br>☐ Abnormal<br>☐ Not Tested	☐ Normal<br>☐ Abnormal<br>☐ Not Tested	☐ Normal<br>☐Abnormal<br>☐ Not Tested	☐ Normal<br>☐Abnormal<br>☐NotTested	☐ Normal<br>☐Abnormal<br>☐NotTested
**Corneal**	☐ Present<br>☐ Absent<br>☐ Not Tested	☐ Present<br>☐ Absent<br>☐ Not Tested	☐ Present<br>☐ Absent<br>☐ Not Tested	☐ Present<br>☐ Absent<br>☐ Not Tested	☐ Present<br>☐ Absent<br>☐ Not Tested	☐ Present<br>☐ Absent<br>☐ Not Tested	☐ Present<br>☐ Absent<br>☐ Not Tested	☐ Present<br>☐ Absent<br>☐ Not Tested	☐ Present<br>☐ Absent<br>☐Not Tested	☐ Present<br>☐ Absent<br>☐NotTested
**Cough**	☐ Normal<br>☐ Abnormal<br>☐ Not Tested	☐ Normal<br>☐ Abnormal<br>☐ Not Tested	☐ Normal<br>☐ Abnormal<br>☐ Not Tested	☐ Normal<br>☐ Abnormal<br>☐ Not Tested	☐ Normal<br>☐ Abnormal<br>☐ Not Tested	☐ Normal<br>☐ Abnormal<br>☐ Not Tested	☐ Normal<br>☐ Abnormal<br>☐ Not Tested	☐ Normal<br>☐ Abnormal<br>☐ Not Tested	☐ Normal<br>☐ Abnormal<br>☐Not Tested	☐ Normal<br>☐ Abnormal<br>☐NotTested
**Gag**	☐ Normal<br>☐ Abnormal<br>☐ Not Tested	☐ Normal<br>☐ Abnormal<br>☐ Not Tested	☐ Normal<br>☐ Abnormal<br>☐ Not Tested	☐ Normal<br>☐ Abnormal<br>☐ Not Tested	☐ Normal<br>☐ Abnormal<br>☐ Not Tested	☐ Normal<br>☐ Abnormal<br>☐ Not Tested	☐ Normal<br>☐ Abnormal<br>☐ Not Tested	☐ Normal<br>☐ Abnormal<br>☐ Not Tested	☐ Normal<br>☐ Abnormal<br>☐ Not Tested	☐ Normal<br>☐ Abnormal<br>☐NotTested
**Swallow**	☐ Normal<br>☐ Abnormal<br>☐ Not Tested	☐ Normal<br>☐ Abnormal<br>☐ Not Tested	☐ Normal<br>☐ Abnormal<br>☐ Not Tested	☐ Normal<br>☐ Abnormal<br>☐ Not Tested	☐ Normal<br>☐ Abnormal<br>☐ Not Tested	☐ Normal<br>☐ Abnormal<br>☐ Not Tested	☐ Normal<br>☐ Abnormal<br>☐ Not Tested	☐ Normal<br>☐ Abnormal<br>☐ Not Tested	☐ Normal<br>☐ Abnormal<br>☐ Not Tested	☐ Normal<br>☐ Abnormal<br>☐NotTested

© 2022 Bell MJ et al. *JAMA Network Open.*

Form Date:_____/_____/ __________ Study ID: -

Entered:_____/____/_________Initials: __________

For office use only

Staff ID: ---------------- _____

ADAPT Trial: PILOT Therapies (PILOT)

Assess every 24 hours. Day 1 begins at time of ICP monitor placement.

Consecutive days begin on the hour (hh:00) of monitor placement.

PICU Day # _____ **Date**_____/_____/__________ **Time**_____: _____

		0	1	2	3
	Scoring →				
**General**	Temperature abnormality (Temp > 38.5ºC requiring cooling, or < 34.5ºC requiring warming)	☐ No	☐ Yes		
**Ventilation**	Status and most frequently observed p_aCO_2 (mmHg) in 24 hr period	☐ Extubated	☐ Intubated, p_aCO_2 > 35	☐ Intubated, p_aCO_2 32-35	Intubated, p_aCO_2 < 32
**CSF Drainage**	Number of times in 24 hrs	☐ None	☐ 0-11 times	12-23 times	≥ 24 times or continuous
**Other Therapies** At any time during 24 hr period	Induced Hypothermia	☐ None	☐ Mild (≥ 35ºC – 37ºC)	Moderate (< 35ºC)	
	Lumbar drain	No	Yes		
	Induced Hypertension (95th %ile for age)	No	Yes		

© 2022 Bell MJ et al. *JAMA Network Open.*

Form Date:_____/_____/___________ Study ID: -

Entered:_____/_____/__________Initials:___________ **For office use only.**

ADAPT: ICU Stay – Physiology (PHYSO)

Staff ID:_____-__ -__ __

Instructions: Pick vital sign closest to the hour. If you have an End Tidal for that hour, use Vitals closest to the End Tidal.

ICU Day #

NOTE THE CPP WILL BE AUTOMATICALLY CALCULATED IN MATRIX

HOUR #	Date mm/dd/yyyy	Time (24-hr clock)		mmHg										ºC				
			HR (Beats/min)	SBP	DBP	MAP	End Tidal CO2	ICP Vent	Highest ICP Vent	ICP IP	Highest ICP IP	CPP	CVP	Temp Brain	Temp Rectal	Temp Esophageal	Temp Bladder	Temp Ax
0																		
1																		
2																		
3																		
4																		
5																		
6																		
7																		
8																		
9																		
10																		
11																		
12																		
13																		
14																		
15																		
16																		
17																		
18																		
19																		
20																		
21																		

© 2022 Bell MJ et al. *JAMA Network Open.*

22																		
23																		

© 2022 Bell MJ et al. *JAMA Network Open.*

Form Date:_____/_____/ __________ Study ID: -

Entered:_____/_____/__________Initials: __________ **For office use only.**

Staff ID ------------------_____

ADAPT:
ICU Stay - Labs (LABS)

ICU Day #

Hour #	Date (mm/dd/yyyy)	Time (24-hr clock)	Platelets # in 1,000s u/L	HGB g/dl	WBC # in 1,000s u/L	NA mmol/L	Serum OSM mOsm/kg	PT seconds	PTT seconds	INR	Phenytoin Levels µg/mL	Phenobarb Level µg/mL	Cortisol µg/dL
0													
1													
2													
3													
4													
5													
6													
7													
8													
9													
10													
11													
12													
13													
14													
15													
16													
17													
18													
19													
20													
21													
22													
23													

© 2022 Bell MJ et al. *JAMA Network Open.*

Form Date:_____/_____/ __________ Study ID: -

Entered:_____/____/_________Initials: _________ **For office use only**

Staff ID ---------------- _____

ADAPT:
Nutrition Labs (NUTR)

ICU Day # ______

Hour #	Date (mm/dd/yyyy)	Time (24-hr clock)	D-stick Glucose (mg/dl)	Serum Glucose (mg/dl)	Cholesterol (mg/dl)	LDL (mg/dl)	VLDL (mg/dl)	Tri – glycerides (mg/dl)	Total Protein (g/dl)	Albumin (g/dl)	Pre-Albumin (mg/dl)
0											
1											
2											
3											
4											
5											
6											
7											
8											
9											
10											
11											
12											
13											
14											
15											
16											
17											
18											
19											
20											
21											
22											
23											

© 2022 Bell MJ et al. *JAMA Network Open.*

Form Date:_____/_____/__________ Study ID: -

Entered:_____/_____/__________Initials:_________ **For office use only**

ADAPT:

Staff ID -------------- ____

Nutritional Support (NUTR2)

ICU Day # _____

Hour #	Date mm/dd/yyyy	Time (24-hr clock)	Parenteral nutrition (TPN) volume (ml)	Dextrose Concentration of TPN (%)	Amino Acid Concentration of TPN (%)	Intralipid volume (ml)	Intralipid Concentration (%)	Enteral nutrition volume (cc)	Enteral nutrition (cal./ oz)	IV Maintenance fluids (cc) 1	Glucose concentration (%) 1	Na Conc (%) 1
0												
1												
2												
3												
4												
5												
6												
7												
8												
9												
10												
11												
12												
13												
14												
15												
16												
17												
18												
19												
20												
21												
22												
23												

© 2022 Bell MJ et al. *JAMA Network Open.*

Form Date:_____/_____/__________ Study ID: -

Hour #	Date mm/dd/yyyy	Time (24-hr clock)	IV Maintenance fluids (cc) 2	Glucose concentration (%) 2	Na Conc (%) 2	Insulin (units)
0						
1						
2						
3						
4						
5						
6						
7						
8						
9						
10						
11						
12						
13						
14						
15						
16						
17						
18						
19						
20						
21						
22						
23						

© 2022 Bell MJ et al. *JAMA Network Open.*

Form Date:_____/_____/ __________ Study ID: -

Entered: / / Initials: **For office use only.**

Staff ID ---------------- _____

ADAPT:
Blood Gases (GAS)

ICU Day #

Hour #	Date (mm/dd/yyyy)	Time (24-hr clock)	PH	PO_2 (mmHg)	PCO_2 (mmHg)	HCO_3 (mMol/L)	Base Excess (mMol/L)	PBO2 (mmHg)	SO2 %	FiO2
0										
1										
2										
3										
4										
5										
6										
7										
8										
9										
10										
11										
12										
13										
14										
15										
16										
17										
18										
19										
20										
21										
22										
23										

© 2022 Bell MJ et al. *JAMA Network Open.*

Form Date:_____/_____/___________ Study ID: -

Entered:_____/_____/__________Initials:_______ **For office use only**

ADAPT:

Staff ID --------------- _____

Hourly Meds (MEDS)

ICU Day # ______

Hour #	Date (mm/dd/yyyy)	Time (24-hr clock)	Hypertonic Saline (cc)	Saline % Concentration	Mannitol (grams)	Mannitol % Concentration	Barbiturates (mg)
0							
1							
2							
3							
4							
5							
6							
7							
8							
9							
10							
11							
12							
13							
14							
15							
16							
17							
18							
19							
20							
21							
22							
23							

© 2022 Bell MJ et al. *JAMA Network Open.*

Form Date:_____/_____/ __________ Study ID: [-]

Entered:_____/____/_________Initials:_______ **For office use only.**

ADAPT:
Hospital Discharge Form (DISCH)

Date of ICU discharge: __ __ /__ __ /__ __ __ __ (mm/dd/yyyy) Time*(use 24-hour clock):*______: _____

Date of hospital discharge(or death): __ __ /__ __ / Time*(use 24-hour clock):*______: _____

mm dd yyyy

Hospital Discharge Destination:

- ☐ Home
- ☐ Rehabilitation facility
- ☐ Skilled nursing facility
- ☐ Other hospital
- ☐ Death → **Complete a "Death" form**
- ☐ Unknown

Consent Information

Was consent obtained for Outcomes?

☐ Yes ☐ No →

Please note reason why consent was not obtained:

☐ Inability to obtain signed consent due to a language barrier.

☐ Refused consent for outcomes. Specify reason:

- ☐ Does not want child to be a research participant
- ☐ Unwilling or unable to travel for outcome testing
- ☐ Research process too burdensome
- ☐ Other, specify reason:_________________________________

____

Enter date consent was obtained:

Date of Consent______/_____/___________
for *outcomes:* mm dd yyyy

→ **Generate the FITBIR GUID and document it below:**

GUID: T B I _______________________

* If consent obtained, complete the Outcomes - Hospital Discharge Packet of forms prior to child's release.

Check if 'Not Fluent in English'

© 2022 Bell MJ et al. *JAMA Network Open.*

↓

☐ Child and parent/guardian are not fluent in English and therefore are not eligible to be tested for part of the 12 month outcome battery.

© 2022 Bell MJ et al. *JAMA Network Open.*

Form Date:_____/_____/ __________ Study ID: -

For office use only

Entered:_____/_____/__________Initials: _______ Time point: ☐0-14 days ☐0-Hosp. Disch

ADAPT:
Medical and Surgical Complications of TBI (COMPLIC)

Staff ID --------------_____

Note: This form will be completed twice (at the end of 14 days and at Hospital Discharge)

1. Were there Systemic Complications ☐ Yes ☐ No (If 'No' Skip to question #2)

Respiratory? ☐ Yes ☐ No

	# of Episodes	*Indicate level of severity of most severe episode.* Mild	Moderate	Severe	Date of Onset of 'Most Severe' episode
ARDS (Acute Respiratory Distress Syndrome)	—	1	2	3	_____ /_____/ ___________
Hemothorax	—	1	2	3	_____ /_____/ ___________
Pneumonia (aspiration)	—	1	2	3	_____ /_____/ ___________
Pneumonia (bacterial/viral/fungal)	—	1	2	3	_____ /_____/ ___________
Pneumothorax	—	1	2	3	_____ /_____/ ___________
Respiratory arrest	—	1	2	3	_____ /_____/ ___________

Cardiovascular? ☐ Yes ☐ No

	# of Episodes	*Indicate level of severity of most severe episode.* Mild	Moderate	Severe	Date of Onset of 'Most Severe' episode
Cardiac Arrest	__	1	2	3	_____ /_____/ ___________
Dysrhythmia	__	1	2	3	_____ /_____/ ___________
Shock	__	1	2	3	_____ /_____/ ___________

General? ☐ Yes ☐ No

	# of Episodes	*Indicate level of severity of most severe episode.* Mild	Moderate	Severe	Date of Onset of 'Most Severe' episode
Acute liver failure	__	1	2	3	_____ /_____/ ___________
Acute renal failure	__	1	2	3	_____ /_____/ ___________
Central line infection	__	1	2	3	_____ /_____/ ___________
Decubitus ulcer	__	1	2	3	_____ /_____/ ___________
Deep vein thrombosis	__	1	2	3	_____ /_____/ ___________
Disseminated intravascular coagulation (DIC)	__	1	2	3	_____ /_____/ ___________
Gastric/Duodenal ulcer	__	1	2	3	_____ /_____/ ___________
Hepatitis	__	1	2	3	_____ /_____/ ___________
Multiple organ dysfunction syndrome	__	1	2	3	_____ /_____/ ___________
Pancreatitis	__	1	2	3	_____ /_____/ ___________
Peritonitis	__	1	2	3	_____ /_____/ ___________

© 2022 Bell MJ et al. *JAMA Network Open.*

Sepsis	__	1	2	3	_____ /_____/ __ - __ __ __

Study ID: -

	# of Episodes	Indicate level of severity of most severe episode.			Date of Onset of 'Most Severe' episode
		Mild	Moderate	Severe	
Septic Shock	__	1	2	3	_____ /_____/ ___________
Wound Infection (non-head)	__	1	2	3	_____ /_____/ ___________

2. Were there Neurological Complications? ☐ Yes ☐ No

	# of Episodes	Indicate level of severity of most severe episode.			Date of Onset of 'Most Severe' episode
		Mild	Moderate	Severe	
CSF leak	__	1	2	3	_____ /_____/ ___________
Diabetes insipidus	__	1	2	3	_____ /_____/ ___________
Herniation syndrome	__	1	2	3	_____ /_____/ ___________
Intracranial abscess	__	1	2	3	_____ /_____/ ___________
Intraparenchymal hemorrhage	__	1	2	3	_____ /_____/ ___________
Intraventricular hemorrhage	__	1	2	3	_____ /_____/ ___________
Meningitis	__	1	2	3	_____ /_____/ ___________
Status Epilepticus	__	1	2	3	_____ /_____/ ___________
Ventriculitis	__	1	2	3	_____ /_____/ ___________
Wound Infection	__	1	2	3	_____ /_____/ ___________
Other, Specify: ______________________	__	1	2	3	_____ /_____/ ___________
Other, Specify: ______________________	__	1	2	3	_____ /_____/ ___________

© 2022 Bell MJ et al. *JAMA Network Open.*

Form Date:______/______/ ___________ Study ID: -

Entered:_____/_____/___________Initials:____________ **For office use only.**

Staff ID ---------------- ____

ADAPT: Radiology Transmission Form (SCANS)

Use this form to document the date and time of image collection andthe date of transfer to the Data Coordinating Center.

Collection:

Date **Time**

_____/_____/__________ _____: _____

Transfer:

Date

_____/_____/__________

© 2022 Bell MJ et al. *JAMA Network Open.*

eTable 3. Complication Definitions

Medical and Surgical Complications of TBI Definitions

Respiratory

ARDS (Acute Respiratory Distress Syndrome) – presence of presence of all three of the following:

(i) bilateral pulmonary infiltrates on chest x-ray, (ii) a PCWP < 18mmHg or no clinical suspicion of leftheart failure and (iii) a consistent (i.e. several hours) PO2: FiO2 ratio of <200

Mild *– N/A*

Moderate *– N/A*

Severe *– all cases classified as severe*

Hemothorax - presence of blood within the plural space that is observed after placement of a chest tube or that is observed by the attending Surgeon during a surgical procedure

Mild *– not requiring therapy*

Moderate *– resolved with placement of chest tube alone without worsening lung disease*

Severe *– resolved with placement of chest tube but also required increased ventilator settings for worsening lung disease*

Pneumonia (aspiration) - Radiographic evidence: 2 or more serial chest radiographs demonstrating at least 1 of the following: (i) new or progressive infiltrate, (ii) persistent infiltrate, (iii) consolidation, (iv) cavitation or pneumatoceles – Plus clinical evidence of at least 1 of the following: fever > 38.5OC, abnormal WBC (<4,000 µl-1, >12,000 µl-1) – Plus clinical signs of at least 2 of the following: new onsetpurulent sputum, change in sputum character, increased respiratory secretions, increased suctioning requirements, worsening cough, worsening dyspnea, rales or bronchial breath sounds, worsening gas exchange (desaturations, increased oxygen requirements or increased ventilator demands) after an incident known to be related to aspiration (vomiting and others) and in the absence of pathological organisms being isolated from respiratory cultures

Mild *– requiring no therapies and no alteration in ventilator parameters*

Moderate *– requiring increases in ventilator settings to maintain oxygenation and ventilation*

Severe *– despite increases in ventilator settings, unable to maintain PaO2 > 60 mm Hg and/or PaCO2 (or end-tidal CO2) < 40 mm Hg*

Pneumonia (bacterial/fungal/viral) - Radiographic evidence: 2 or more serial chest radiographs demonstrating at least 1 of the following: (i) new or progressive infiltrate, (ii) persistent infiltrate, (iii) consolidation, (iv) cavitation or pneumatoceles – Plus clinical evidence of at least 1 of the following: fever > 38.5^{O}C, abnormal WBC (<4,000 µl-1, >12,000 µl-1) – Plus clinical signs of at least 2 of the following: new onset purulent sputum, change in sputum character, increased

© 2022 Bell MJ et al. *JAMA Network Open.*

respiratory secretions,increased suctioning requirements, worsening cough, worsening dyspnea, rales or bronchial breath sounds, worsening gas exchange (desaturations, increased oxygen requirements or increased ventilator demands) with the isolation of a pathological organism in a respiratory culture

Mild *– requiring no therapies and no alteration in ventilator parameters*

Moderate *– requiring increases in ventilator settings to maintain oxygenation and ventilation*

Severe *– despite increases in ventilator settings, unable to maintain PaO2 > 60 mm Hg and/or PaCO2 (or end-tidal CO2) < 40 mm Hg*

Pneumothorax – presence of air within the pleural space as diagnosed by imaging studies from the attending Surgeon or Radiologist

Mild *– not requiring therapy*

Moderate *– resolved with placement of chest tube alone without worsening lung disease*

Severe *– resolved with placement of chest tube but also required increased ventilator settings for worsening lung disease*

Respiratory arrest – cessation of breathing sufficient to require bag-valve mask or other artificialapparatus to support pulmonary function

Mild *– N/A*

Moderate *– N/A*

Severe *– all cases classified as severe*

Cardiovascular

Cardiac arrest – cessation of heart function (rhythm and blood pressure) sufficient to requirecardiopulmonary resuscitation (including chest compressions)

Mild *– N/A*

Moderate *– N/A*

Severe *– all cases classified as severe*

Dysrhythmia – abnormal heart rhythm consisting of any of the following all of which lasting at least 30seconds and confirmed by a pediatric cardiologist:

1. Narrow-complex tachycardia with absent or abnormal P waves (different morphology oraxis) with normal QRS complex
2. Wide-complex tachycardia with widened QRS complex, may be associated with

© 2022 Bell MJ et al. *JAMA Network Open.*

inverted Twave axis

3. Sustained bradycardia of less than 60 beats/min if age < 6 years, < 45 for age 7- 11 years, <40 for age > 11 years

Mild *– requires no treatment for resolution*

Moderate *– requires medical therapy for resolution, no associated cardiovascular compromise duringthe event*

Severe *– requires medical therapy for resolution but associated with cardiovascular compromise during the event*

Cardiogenic Shock - Cardiogenic shock is defined by sustained hypotension requiring cardiopressor therapy resulting from inadequate circulation of blood, due to primary failure of the ventricles of the heart to function effectively

Mild *– N/A*

Moderate *– N/A*

Severe *– all cases classified as severe*

General

Acute liver failure - total bilirubin > 4 mg/dl OR ALT/AST > 2SD above aged-norm

Mild *– requiring no therapy*

Moderate *– requiring transfusion of fresh frozen plasma for correction of coagulopathy or any othermedical therapy for liver dysfunction*

Severe *– requiring invasive therapies (plasma exchange, artificial liver preparations, etc) or life-threatening*

Acute renal failure - Oliguria (urine output < 0.5 ml/kg/h x 12 h with adequate CVP) or creatinine (increased by 0.5 md/dl or greater than 2SD from aged-based norm)

Mild *– requiring no therapy*

Moderate *– N/A*

Severe *– requiring dialysis*

Central line infection - isolation of pathogenic bacteria from blood culture from an indwelling catheterwith negative blood cultures from other sites (either peripheral venipuncture or other indwelling catheters), not believed to be contaminated from the processing of the specimen

Mild *– requiring no therapy*

© 2022 Bell MJ et al. *JAMA Network Open.*

Moderate *– requiring antibiotic therapy*

Severe *– requiring removal of the catheter because of persistent positive cultures*

Decubitus ulcer - Development of skin breakdown over dependent region of the back, extremity, torsoor head with erythema or greater disruption of skin integrity

Mild *– requiring no therapy*

Moderate *– requiring occlusive dressing or other non-invasive therapies*

Severe *– requiring surgical debridement*

Deep vein thrombosis – presence of a clot (diagnosed by a radiologist) within one of the great vessels (jugular, subclavian, axillary, femoral, iliac, inferior vena cava or superior vena cava)

Mild *– requiring no therapy*

Moderate *– requiring anticoagulation therapy*

Severe *– requiring anticoagulation therapy plus a surgical procedure (thrombectomy, placement of filteror other procedure)*

DIC (Disseminated intravascular coagulation) – evidence of activation of the coagulation system by the following: persistent coagulopathy (INR > 1.5 or aPTT > 60 sec), thrombocytopenia (platelet count < 100,000 µl-1)

Mild *– requiring no therapy*

Moderate *– requiring replacement of fresh frozen plasma and/or platelets*

Severe *– evidence of hemorrhage that is believed to be due to the syndrome*

Gastric/Duodenal ulcer - presence of melena or occult blood per rectum (must be heme-test positive) OR presence of coffee grounds or blood by NG/oral (must be heme-test positive) OR documentation of bleeding via endoscopy

Mild *– requiring no therapy*

Moderate *– requiring cessation of feeds and/or blood transfusion*

Severe *– requiring endoscopy for sufficient hemostasis or any surgical procedure*

Hepatitis - inflammation of the liver with a total bilirubin >4mg/dL or ALT/AST > 2SD above aged–norm

Mild *- Requiring supportive care only, fluids, rest, pain medication*

© 2022 Bell MJ et al. *JAMA Network Open.*

Moderate *- Above plus needing medical intervention such as antiviral medications*

Severe *- Above plus acute liver failure*

Multiple Organ Dysfunction Syndrome – meeting definition of SIRS (below)

SIRS - More than 2 of the following:

Tachycardia (HR > 2SD from norm);Tachypnea (RR > 2SD from norm);
Leukocyte abnormality (WBC < 4,000 or >12,000 µl-1; or more than 10%immature forms with normal WBC count);
CRP > 2SD above norm for age;Decreased capillary refill

AND at least 1 of the following

Hypotension (MAP < 2SD from norm);Hypoxemia (P/F ratio < 300);
Renal dysfunction (oliguria (urine output < 0.5 ml/kg/h x 2 h) or Cr (increase > 0.5mg/dl));
Coagulopathy (INR > 1.5 or aPTT > 60 sec);
Liver dysfunction (plasma total bilirubin > 4 mg/dl);Thrombocytopenia (platelet count < 100,000 µl-1);Hyperlactatemia (>1 mmol/L)
Altered mental status

Plus, at least 3 of the following:

Hypotension: MAP < 2SD from norm requiring vasopressor support;Hypoxemia: P/F ratio < 300 or any need for mechanical ventilation;
Renal failure/dysfunction: (oliguria (urine output < 0.5 ml/kg/h x 12 h) or Cr(increase > 0.5 mg/dl));
Coagulopathy: INR > 1.5 or aPTT > 60 sec;
Liver failure/dysfunction: plasma total bilirubin > 4 mg/dl;
Thrombocytopenia/hematological failure: platelet count < 100,000 µl-1;Hyperlactatemia (>1 mmol/L)
Altered mental status (assuming this is not from the TBI)

Mild *– N/A*

Moderate *– N/A*

Severe *– all cases classified as severe*

Pancreatitis **-** Increase in serum markers of pancreatitis (amylase or lipase) greater than 2SD above

© 2022 Bell MJ et al. *JAMA Network Open.*

laboratory normal for individual institutions or demonstration of pancreatic pseudocyst diagnosed by a radiologist

Mild *– requiring no therapy*

Moderate *– necessitating alteration of feeding regimen*

Severe *– concomitant presence of shock or other organ failure*

<u>Peritonitis</u> - recovery of pathogenic organism from ascetic fluid or clinical signs (fever >38.5 degreesC, rigid abdomen, absence of bowel sounds, presence of fluid wave) PLUS WBC (>1000/ml from peritoneal lavage.

Mild *- recovery of pathogenic organism from ascetic fluid or clinical signs (fever >38.5 degrees C, rigidabdomen, absence of bowel sounds, presence of fluid wave) PLUS WBC (>1000/ml from peritoneal lavage requiring general supportive measures such as intravenous rehydration and correction of electrolyte disturbances and broad spectrum antibiotics*

Moderate *– recovery of pathogenic organism from ascetic fluid OR clinical signs (fever > 38.5 degrees C, rigid abdomen, absence of bowel sounds, presence of fluid wave) PLUS WBC (>1000/ml) from peritoneal lavage SAE requiring surgical intervention associated with sepsis.*

Severe *- recovery of pathogenic organism from ascetic fluid OR clinical signs (fever > 38.5 degrees C, rigid abdomen, absence of bowel sounds, presence of fluid wave) PLUS WBC (>1000/ml) from peritoneal lavage SAE requiring surgical intervention or associated with septic shock and/or organ failure*

<u>Sepsis</u> - Infection (documented or clinically suspected) <u>plus</u> recovery of pathogenic organism from anormally sterile site OR isolation of bacteria from a site where the bacteria is not normally located (example: isolation of Enterococcus from nasal sinus) PLUS meet the definition of SIRS (below)

SIRS - More than 2 of the following:

Tachycardia (HR > 2SD from norm);Tachypnea (RR > 2SD from norm);
Leukocyte abnormality (WBC < 4,000 or >12,000 µl-1; or more than 10%immature forms with normal WBC count);
CRP > 2SD above norm for age;Decreased capillary refill

AND at least 1 of the following

Hypotension (MAP < 2SD from norm);Hypoxemia (P/F ratio < 300);
Renal dysfunction (oliguria (urine output < 0.5 ml/kg/h x 2 h) or Cr (increase >

© 2022 Bell MJ et al. *JAMA Network Open.*

0.5mg/dl));
Coagulopathy (INR > 1.5 or aPTT > 60 sec);
Liver dysfunction (plasma total bilirubin > 4 mg/dl); Thrombocytopenia (platelet count < 100,000 µl-1); Hyperlactatemia (>1 mmol/L)
Altered mental status

Mild *– meeting the definition of SIRS (based on above criteria)*

Moderate *– meeting the definition of Sepsis (2 SIRS criteria plus confirmed or suspected infection)*

Severe *– meeting the definition of Sepsis plus end organ dysfunction*

Septic Shock – see above with the addition of needs to require vasopressor medications to maintain blood pressure

Mild *– N/A*

Moderate *– N/A*

Severe *– all cases classified as severe*

Wound infection (non-head) **-** recovery of pathogenic organism from a normally sterile site or isolationof bacteria from a site where the bacteria is not normally located; site must not be on the head

Mild *– no clinical symptoms*

Moderate *– clinical symptoms of erythema and fever, requiring antibiotics*

Severe *– clinical symptoms above, plus the need for surgical debridement*

Neurological

CSF leak **-** Leakage of cerebrospinal fluid, as confirmed by lab report positive for Beta-2 transferrin from drainage site (ventriculotomy or lumbar drain) or other body sites (nose, ears, or other locations)

Mild *– no clinical symptoms and requiring no therapies*

Moderate *– requires non-surgical therapy (placement of lumbar drain, antibiotic therapy, etc)*

Severe *– requires surgical correction*

Diabetes Insipidus – in the absence of diuretics, an acute increase in urine output associated with sustained increases in serum sodium with low urine sodium/low urine specific gravity

Mild *– no clinical symptoms and requiring no therapies*

© 2022 Bell MJ et al. *JAMA Network Open.*

***Moderate** – requires adjustments of sodium concentrations in intravenous fluid solutions*

***Severe** – requires treatment with vasopressin infusion or DDAVP*

<u>Herniation Syndrome</u> - clinical findings including fixed/dilated pupil(s), apnea, bradycardia and/orhypertension plus CT/MRI evidence of herniation as identified by attending Neurosurgeon or Neuroradiologist

***Mild** – N/A*

***Moderate** – N/A*

***Severe** – all cases will be categorized as severe*

<u>Intracranial abscess</u> - presence of space-occupying lesion with purulent material within the brain parenchyma as evidence by CT/MR in the opinion of the attending Neurosurgeon or Neuroradiologist

***Mild** – N/A*

***Moderate** – resolved with medical therapy alone*

***Severe** – require surgical procedure to alleviate*

<u>Intraparenchymal hemorrhage</u> - presence of blood within the brain parenchyma as evidence byCT/MR in the opinion of the attending Neurosurgeon/Neuroradiologist

***Mild** – incidental finding on imaging that results in no clinical symptoms*
***Moderate** – findings on imaging sufficient to cause some clinical symptoms**Severe** – require surgical procedure to alleviate*

<u>Intraventricular hemorrhage</u> - presence of blood within the intraventricular space as evidenced byCT/MR in the opinion of the attending Neurosurgeon/Neuroradiologist

***Mild** – incidental finding on imaging that results in no clinical symptoms*
***Moderate** – findings on imaging sufficient to cause some clinical symptoms**Severe** – require surgical procedure to alleviate*

<u>Meningitis</u> - pathological organisms isolated from the CSF obtained from a lumbar puncture or lumbardrain, not believed to be contaminated by the processing of the specimen **PLUS** abnormal CSF (increased WBC, increased protein, decreased glucose, organisms seen on gram stain)

***Mild** - no clinical symptoms*

***Moderate** – clinical symptoms of headache, neck stiffness and/or fever*

© 2022 Bell MJ et al. *JAMA Network Open.*

Severe *– signs of neurological compromise (new neurological findings including but not limited to newfocal signs/symptoms, new strokes and others)*

Status epilepticus - observation of a clinically-apparent seizure lasting at least 5 minutes by an MD without return to neurological baseline or electrographic evidence of seizures for more than 5 minutesas interpreted by a pediatric neurologist

Mild *– resolution of events without treatment*

Moderate *– resolution of events with treatment with administration of bolus medications*

Severe *– resolution of events requires continuous infusions of medications or events do not resolve*

Ventriculitis - pathological organisms isolated from the CSF obtained from a ventricular catheter, not believed to be contaminated by the processing of the specimen plus abnormal CSF (increased WBC, increased protein, decreased glucose, organisms seen on gram stain)

Mild *- no clinical symptoms*

Moderate *– clinical symptoms of headache, neck stiffness and/or fever*

Severe *– signs of neurological compromise (new neurological findings including but not limited to new focal signs/symptoms, new strokes and others)*

Wound infection - recovery of pathogenic organism from a normally sterile site or isolation of bacteriafrom a site where the bacteria is not normally located; site must be on the head

Mild *– no clinical symptoms*

Moderate *– clinical symptoms of erythema and fever, requiring antibiotics*

Severe *– clinical symptoms above, plus the need for surgical debridement*

Other - other neurological complications

Mild *– resolved without interventions*

Moderate *– required interventions to resolve*

Severe *– life-threatening or prolonged resolution*

© 2022 Bell MJ et al. *JAMA Network Open.*

eFigure 1. Kaplan-Meier Time to Death

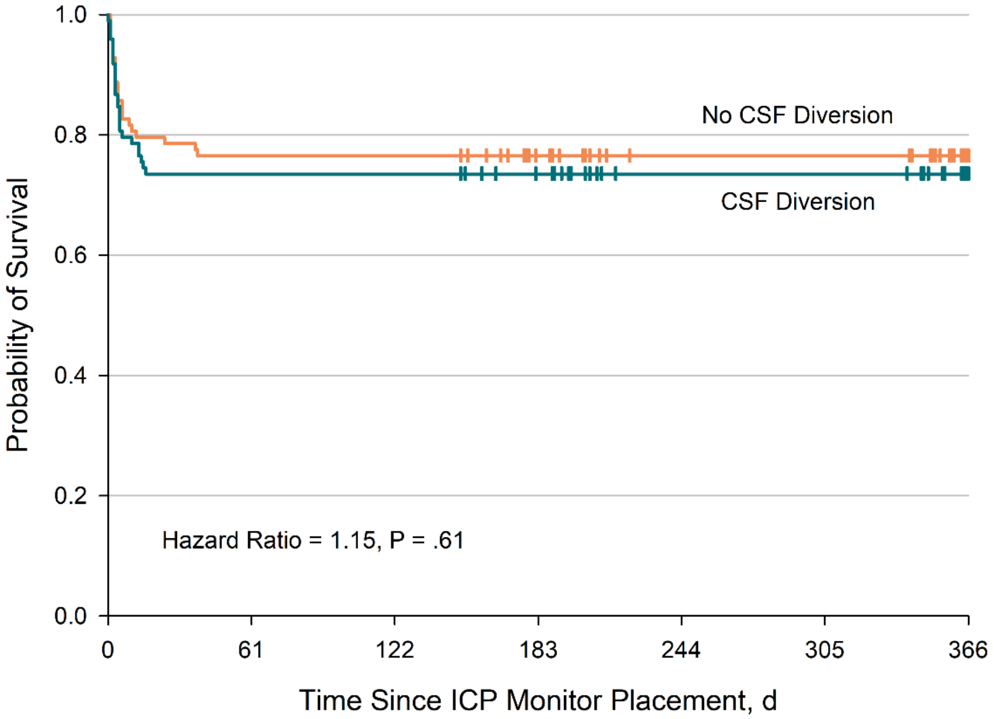

N at Risk							
CSF Diversion	98	72	72	67	53	53	38
No CSF Diversion	98	75	75	64	55	55	38

© 2022 Bell MJ et al. *JAMA Network Open.*

eTable 4. Sensitivity Analysis for Delayed CSF Diversion

Sensitivity Analyses of Primary and Secondary Outcomes,

	Matched Patients (N=105 Pairs)[a]			Matched Patients (N=146 Pairs)[e]		
	**Median Difference**	**Quartile 1, Quartile 3**	**P**	**Median Difference**	**Quartile 1, Quartile 3**	**P**
**Primary**						
GOS—E Peds[b]	0	-2, 3	.09	0	-2, 3	.95
	**Odds Ratio/ Hazard Ratio**	**95% Confidence Interval**	**P**	**Odds Ratio/ Hazard Ratio**	**95% Confidence Interval**	**P**
**Secondary**						
Death[c]	1.44	(0.76-2.72)	.26	1.11	(0.66-1.84)	.70
Time to death[d]	1.34	(0.77-2.33)	.30	1.09	(0.71-1.67)	.70
Complications[c]						
Respiratory	0.70	(0.39-1.26)	0.75	(0.41-1.38)	.36	
Cardiovascular	1.51	(0.61-3.69)	1.35	(0.46-4.00)	.58	
General	1.00	(0.49-2.02)	1.13	(0.56-2.28)	.72	
Neurological	1.48	(0.85-2.58)	0.83	(0.49-1.42)	.49	

Abbreviations: GOS—E Peds, Glasgow Outcome Scale—Extended Pediatric Version.

[a] A total of 62 children received CSF diversion after the initial ICP monitor was placed and were included in the CSF diversion group.

[b] Wilcoxon signed-rank test.

[c] Odds ratio.

[d] Hazard ratio.

[e] A total of 146 children received CSF diversion after the initial ICP monitor was placed and were included in the CSF diversion group.

© 2022 Bell MJ et al. *JAMA Network Open.*

eFigure 2. Consort Diagram for Sensitivity Analysis

eFigure 2. CONSORT Diagram for Sensitivity Analysis

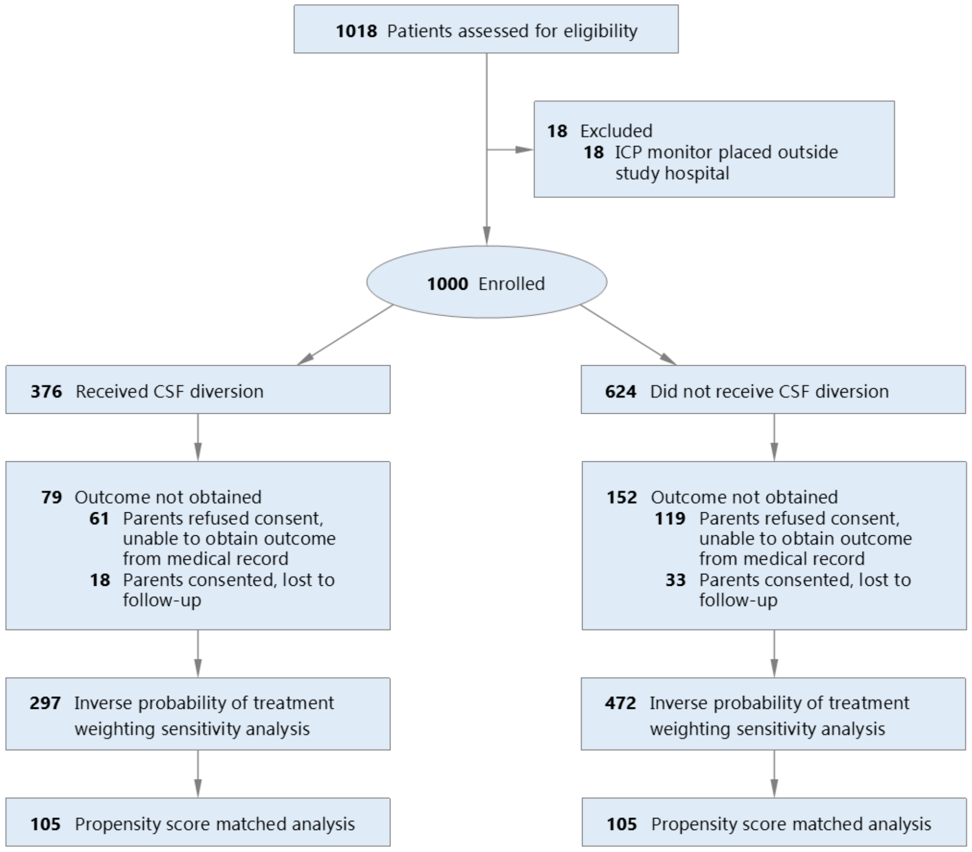

Subjects were stratified based on decisions regarding CSF diversion and were enrolled prior to obtaining consent for outcomes. CSF denotes cerebrospinal fluid.

© 2022 Bell MJ et al. *JAMA Network Open.*

eFigure 3. Classification Tree and Boxplots of Propensity Scores to Exclude Subgroups Unlikely to Receive CSF Diversion for Sensitivity Analysis

A. Classification Tree

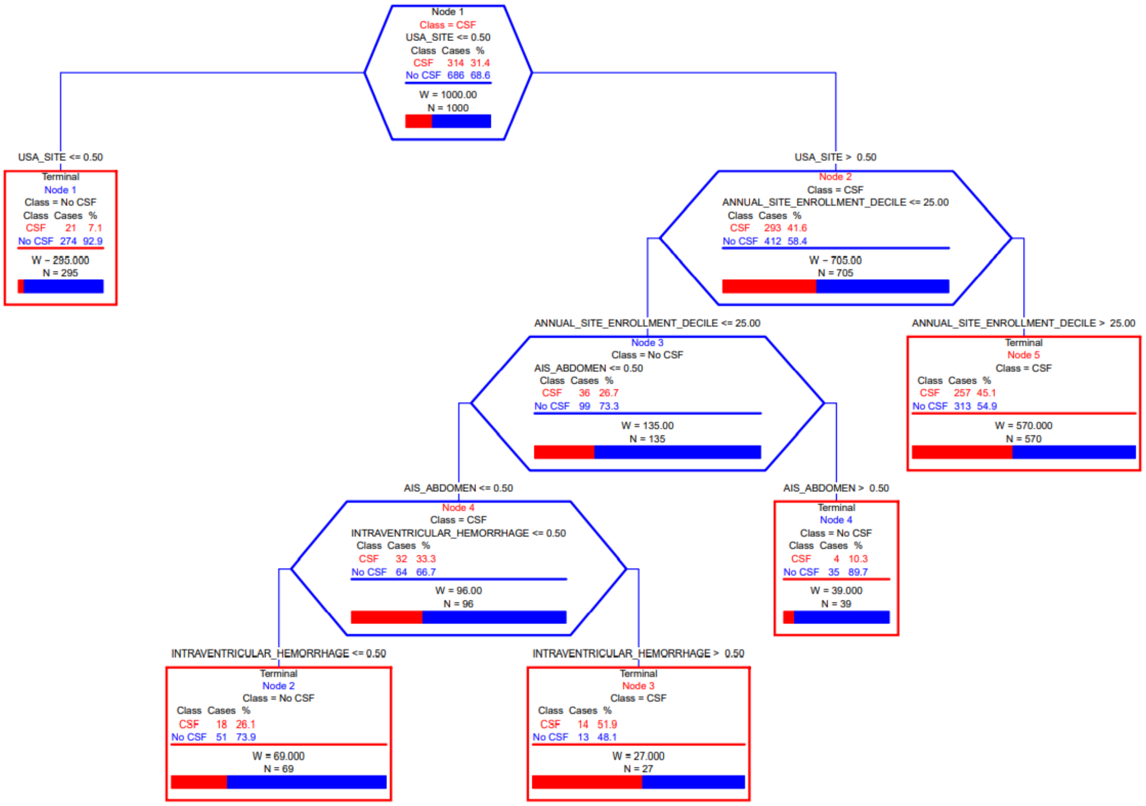

© 2022 Bell MJ et al. *JAMA Network Open.*

B. Boxplots

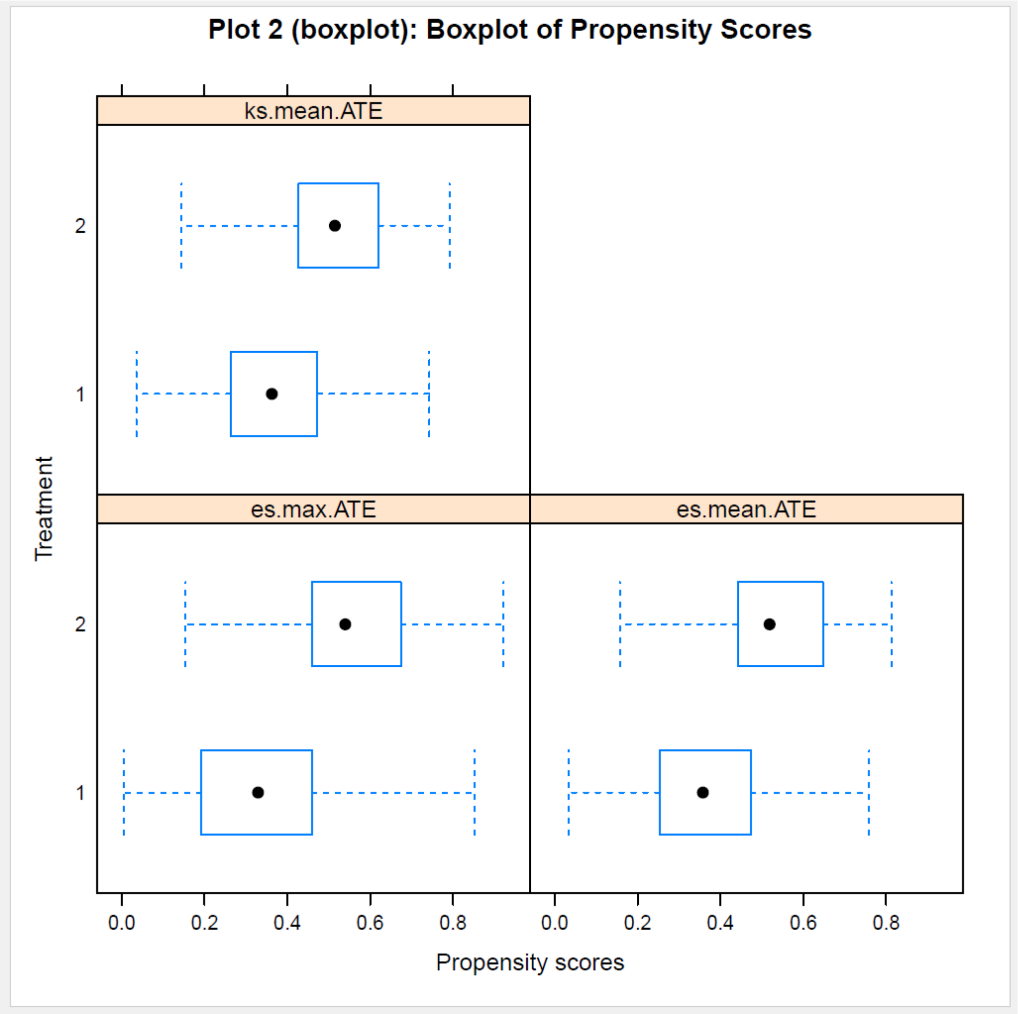

© 2022 Bell MJ et al. *JAMA Network Open.*
