# Supplementary material for: Comparative Effectiveness of Diversion of Cerebrospinal Fluid for Children With Severe Traumatic Brain Injury
Source: JAMA Netw Open. 2022 Jul 8;5(7):e2220969. doi: 10.1001/jamanetworkopen.2022.20969 (PMC9270700; doi:10.1001/jamanetworkopen.2022.20969)
Supplement: Supplement 2. — The Approaches and Decisions for Acute Pediatric TBI (ADAPT) Investigators [file jamanetwopen-e2220969-s002.pdf]

Supplemental Online Content: Nonauthor Collaborators

*Indicates required information. Only first name, last name, and suffix will appear in PubMed.

***Group Name(s): Approaches and Decisions for Acute Pediatric TBI (ADAPT) Investigators**							
***First Name and Middle Initial(s)**	***Last Name**	***Suffix (eg, Jr, III)**	Academic Degrees	Institution	Location (city, state/province, country)	Role or Contribution, eg, chair, principal investigator	Group (if more than 1 Group listed in the byline) and/or Subgroup (eg, Steering Committee)
Shruti	Agarwal		MD	Addenbrookes Hospital	Cambridge UK	Site investigator	
Sarah	Mahoney		MD	Alder Hey Children's Hospital	Liverpool UK	Site investigator	
John	Beca		MD	Starship Children's Hospital	Auckland NZ	Site investigator	
Laura	Loftis		MD	Texas Children's Hospital	Houston TX	Site investigator	
Lauren	Piper		MD	Levine Children's Hospital	Charlotte NC	Site investigator	
Anthony	Slater		MD	Children's Health Queensland Hospital and Health Service	Brisbane AU	Site investigator	
Karen	Walson		MD	Children's Health Care of Atlanta	Atlanta GA	Site investigator	
Tellen	Bennett		MD	Children's Hospital of Colorado	Aurora CO	Site investigator	
Todd	Kilbaugh		MD	Children's Hospital of Philadelphia	Philadelphia PA	Site investigator	
Iqbal	O'Meara		MD	Children's Hospital of Richmond	Richmond VA	Site investigator	
Nikki	Miller Ferguson		MD	Children's Hospital of Richmond	Richmond VA	Site investigator	
Ranjit	Chima		MD	Children's Hospital of Cincinnati	Cincinnati OH	Site investigator	
Brad	Kurowski		MD	Children's Hospital of Cincinnati	Cincinnati OH	Site investigator	
Enno	Wildshut		MD	Erasmus Medical Center	Rotterdam Netherlands	Site investigator	
Naomi	Ketharanathan		MD	Erasmus Medical Center	Rotterdam Netherlands	Site investigator	
Mark	Peters		MD	Great Ormond Street	London UK	Site investigator	
Kerri	LaRovere		MD	Boston Children's Hospital	Cambridge MA	Site investigator	
Robert	Tasker		MD	Boston Children's Hospital	Cambridge MA	Site investigator	
Joan	Balcells		MD	Hospital Vall d'Hebron	Barcelona, Spain	Site investigator	
Courtney	Robertson		MD	Johns Hopkins University	Baltimore MD	Site investigator	
Akash	Deep		MD	Kings College Hospital	London UK	Site investigator	
Sian	Cooper		MD	Leeds Teaching Hospitals	Leeds UK	Site investigator	
Sarah	Murphy		MD	Massachussetts General Hospital	Boston MD	Site investigator	
John	Kuluz		MD	Miami Children's Hospital	Miami FL	Site investigator	
Nicole	O'Brien		MD	Nationwide Children's Hospital	Columbus OH	Site investigator	
Neal	Thomas		MD	Pennsylvania State University	Hershey PA	Site investigator	
Frederick	Willyerd		MD	Phoenix Children's Hospital	Phoenix AZ	Site investigator	

Supplemental Online Content: Nonauthor Collaborators

*Indicates required information. Only first name, last name, and suffix will appear in PubMed.

***First Name and Middle Initial(s)**	***Last Name**	***Suffix (eg, Jr, III)**	Academic Degrees	Institution	Location (city, state/province, country)	Role or Contribution, eg, chair, principal investigator	Group (if more than 1 Group listed in the byline) and/or Subgroup (eg, Steering Committee)
Simon	Erickson		MD	Perth Children's Hospital	Perth AU	Site investigator	
J. Mahil	Samuel		MD	Royal Manchester Children's Hospital	Manchester UK	Site investigator	
Rachel	Agbeko		MD	Newcastle on Tyne Foundation Trust	Newcastle UK	Site investigator	
Iain	Macintosh		MD	University Hospital Southampton	Southampton UK	Site investigator	
Michele	Kong		MD	University of Alabama Birmingham	Birmingham AL	Site investigator	
Joanne	Natale		MD	University of California Davis	Sacramento CA	Site investigator	
Heather	Siefkes		MD	University of California Davis	Sacramento CA	Site investigator	
Christopher	Giza		MD	University of California, Los Angeles	Los Angeles CA	Site investigator	
Hari	Thangarajah		MD	University of California San Diego	San Diego CA	Site investigator	
David	Shellington		MD	University of California San Diego	San Diego CA	Site investigator	
Elizabeth	Newell		MD	University of Iowa	Iowa City IA	Site investigator	
Christopher	Giza		MD	University of California Los Angeles	Los Angeles CA	Site investigator	
Edward	Truemper		MD	University of Nebraska	Omaha NE	Site investigator	
Sidharth	Mahapatra		MD	University of Nebraska	Omaha NE	Site investigator	
Robert	Clark		MD	University of Pittsburgh	Pittsburgh PA	Site investigator	
Jamie	Hutchison		MD	Hospital for Sick Children	Toronto CA	Site investigator	
Nadeem	Shafi		MD	LeBonheur Children's Hospital	Memphis TN	Site investigator	
Alino Nico	West		PhD	LeBonheur Children's Hospital	Memphis TN	Site investigator	
Darryl	Miles		MD	University of Texas Southwestern	Dallas TX	Site investigator	
Mark	Wainwright		MD	University of Washington	Seattle WA	Site investigator	
Monica	Vavilala		MD	University of Washington	Seattle WA	Site investigator	
Peter	Ferrazzano		MD	University of Wisconsin	Madison WI	Site investigator	
Stuart	Friess		MD	Washington University of St. Louis	St. Louis MO	Site investigator	
Ajit	Sarnaik		MD	Wayne State University	Detroit MI	Site investigator	
